# An engineered CRISPR–Cas12i tool for efficient multiplexed genome editing

**DOI:** 10.1093/nar/gkaf806

**Published:** 2025-08-27

**Authors:** Linli Wang, Yanlu Wang, Jian Chen, Yaning Zhu, Hao Qin, Jie Liu, Yue Ai, Jinsheng Lai, Zhengxing Lian, Hongbing Han

**Affiliations:** State Key Laboratory of Animal Biotech Breeding, China Agricultural University, Beijing 100193, China; Beijing Key Laboratory of Animal Genetic Improvement, College of Animal Science and Technology, China Agricultural University, Beijing 100193, China; Key Laboratory of Animal Genetics, Breeding and Reproduction of the Ministry of Agriculture and Rural Affairs, College of Animal Science and Technology, China Agricultural University, Beijing 100193, China; State Key Laboratory of Animal Biotech Breeding, China Agricultural University, Beijing 100193, China; Beijing Key Laboratory of Animal Genetic Improvement, College of Animal Science and Technology, China Agricultural University, Beijing 100193, China; Key Laboratory of Animal Genetics, Breeding and Reproduction of the Ministry of Agriculture and Rural Affairs, College of Animal Science and Technology, China Agricultural University, Beijing 100193, China; Frontiers Science Center for Molecular Design Breeding (MOE), China Agricultural University, Beijing 100193, China; State Key Laboratory of Animal Biotech Breeding, China Agricultural University, Beijing 100193, China; Beijing Key Laboratory of Animal Genetic Improvement, College of Animal Science and Technology, China Agricultural University, Beijing 100193, China; Key Laboratory of Animal Genetics, Breeding and Reproduction of the Ministry of Agriculture and Rural Affairs, College of Animal Science and Technology, China Agricultural University, Beijing 100193, China; State Key Laboratory of Animal Biotech Breeding, China Agricultural University, Beijing 100193, China; Beijing Key Laboratory of Animal Genetic Improvement, College of Animal Science and Technology, China Agricultural University, Beijing 100193, China; Key Laboratory of Animal Genetics, Breeding and Reproduction of the Ministry of Agriculture and Rural Affairs, College of Animal Science and Technology, China Agricultural University, Beijing 100193, China; State Key Laboratory of Animal Biotech Breeding, China Agricultural University, Beijing 100193, China; Beijing Key Laboratory of Animal Genetic Improvement, College of Animal Science and Technology, China Agricultural University, Beijing 100193, China; Key Laboratory of Animal Genetics, Breeding and Reproduction of the Ministry of Agriculture and Rural Affairs, College of Animal Science and Technology, China Agricultural University, Beijing 100193, China; State Key Laboratory of Animal Biotech Breeding, China Agricultural University, Beijing 100193, China; Beijing Key Laboratory of Animal Genetic Improvement, College of Animal Science and Technology, China Agricultural University, Beijing 100193, China; Key Laboratory of Animal Genetics, Breeding and Reproduction of the Ministry of Agriculture and Rural Affairs, College of Animal Science and Technology, China Agricultural University, Beijing 100193, China; Frontiers Science Center for Molecular Design Breeding (MOE), China Agricultural University, Beijing 100193, China; State Key Laboratory of Animal Biotech Breeding, China Agricultural University, Beijing 100193, China; Beijing Key Laboratory of Animal Genetic Improvement, College of Animal Science and Technology, China Agricultural University, Beijing 100193, China; Key Laboratory of Animal Genetics, Breeding and Reproduction of the Ministry of Agriculture and Rural Affairs, College of Animal Science and Technology, China Agricultural University, Beijing 100193, China; Frontiers Science Center for Molecular Design Breeding (MOE), China Agricultural University, Beijing 100193, China; State Key Laboratory of Animal Biotech Breeding, China Agricultural University, Beijing 100193, China; Beijing Key Laboratory of Animal Genetic Improvement, College of Animal Science and Technology, China Agricultural University, Beijing 100193, China; Key Laboratory of Animal Genetics, Breeding and Reproduction of the Ministry of Agriculture and Rural Affairs, College of Animal Science and Technology, China Agricultural University, Beijing 100193, China

## Abstract

Developing efficient and simplified tools for multiplexed genome editing remains challenging due to limitations in precursor CRISPR RNA (pre-crRNA) processing and reliance on additional RNA-based regulatory components. Cas12i.3, a small RNA-guided nuclease, reportedly lacks pre-crRNA processing ability, restricting its multiplexing capability. Here, we engineered Cas12i.3 by optimizing CRISPR RNA (crRNA) design, codon usage, and exonuclease fusion, generating initial optimized Cas12i (IOCas12i) system. Further rational design and amino acid mutations yielded the highly efficient enhanced optimized Cas12i (EOCas12i) systems, EOCas12i–Combo1 and EOCas12i–Combo2, exhibiting 2.5- to 22.8-fold and 3.0- to 60.0-fold editing efficiencies relative to wild-type Cas12i.3, comparable to *Streptococcus pyogenes* Cas9 (SpCas9) and Lachnospiraceae bacterium Cas12a (LbCas12a). Additionally, they exhibited high specificity and produced longer insertions and deletions (indels) that may facilitate gene knockout. Notably, both variants enabled efficient multiplexed editing of up to 30 targets using compact crRNA arrays. These advancements position EOCas12i–Combo1 and EOCas12i–Combo2 as promising platforms for multiplexed genome editing applications.

## Graphical Abstract

**Graphical Abstract:**
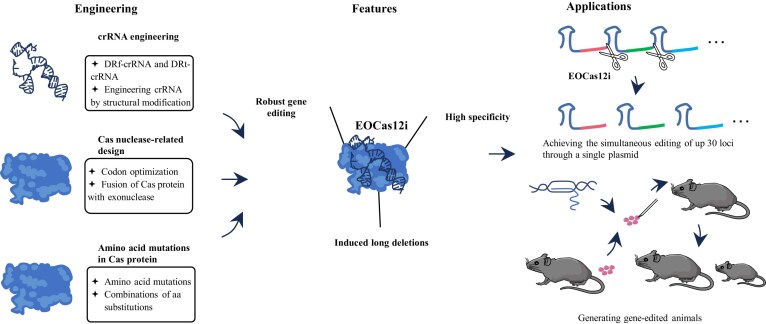

## Introduction

Higher organisms utilize complex genetic networks with functionally redundant genes to fine-tune cellular processes. Consequently, tools capable of manipulating multiple genes simultaneously are invaluable for both basic research and practical applications of genetic engineering. Several CRISPR–Cas (clustered regularly interspaced short palindromic repeats and CRISPR-associated proteins) effectors, including *Streptococcus pyogenes* Cas9 (SpCas9), *Alicyclobacillus acidoterrestris* Cas12b (AaCas12b), *Acidaminococcus*sp. Cas12f (AsCas12f), and Lachnospiraceae bacterium Cas12g (LbCas12g), exhibit nuclease activity but lack the ability to independently process precursor CRISPR RNA (pre-crRNA) or do not function without *trans*-activating CRISPR RNA (tracrRNA) [1]. As a result, either the presence of long RNA-based regulatory elements (e.g. transfer RNAs (tRNAs) and ribozymes) or the heterologous expression of additional proteins (e.g. Csy4) are required to enable regulation of multiple genes using current platforms, making them less suitable for efficient multiplexed genome editing [2–4].

Cas12i.3 is characterized by a relatively small size (1045 amino acids) compared with SpCas9 (1368 amino acids) and AsCas12a (1307 amino acids), with simple protospacer adjacent motif (PAM) requirements (5′-TTN). However, its application has been limited, as existing reports in plants indicate relatively low editing efficiency [5]. Although protein mutations have been explored to enhance Cas12i.3’s editing efficiency [6], other critical optimization strategies—such as CRISPR RNA (crRNA) design [7–10], codon optimization [11], and exonuclease fusion [12]—remain largely underexplored despite their proven impact on editing performance. Cas12a has been proven effective for multiplexed genome editing; however, reports indicate that Cas12i.3, from the same type V family, lacks pre-crRNA processing capability in plants when unoptimized [13, 14]. Given the similarities between Cas12i.3 and Cas12a, a systematic optimization of Cas12i.3’s editing efficiency could potentially overcome this limitation, making it a more compact and promising tool for multiplex gene editing.

In this study, we optimized CRISPR–Cas12i.3 through enhancing crRNA design, incorporating codon optimization and exonuclease fusion into the Cas12i.3 protein. These modifications, targeting both crRNA and Cas12i.3 protein, were integrated to generate initial optimized Cas12i (IOCas12i) system. Building on IOCas12i, we applied rational design and amino acid mutations, followed by regional combinations, to develop the highly efficient enhanced optimized Cas12i (EOCas12i) systems. EOCas12i shows superior efficiency and specificity compared to widely used Cas nucleases and is suitable for multiplexed genome editing. Therefore, our newly engineered EOCas12i systems represent a promising platform for multiplexed genome editing.

## Materials and methods

### Animal ethics statement

All animal experiments were approved and supervised by the Animal Welfare Committee of China Agricultural University (Approval ID: AW72205202-1-01).

### Plasmid construction

Mammalian codon-optimized Cas12i.3 with nuclear localization signal, linker, Triplex, 20-target array, 30-target array, and T5 exonuclease (T5E) were synthesized by the GeneWiz Co., Ltd. and incorporated into a mammalian expression vector under the CBh promoter by Gibson Assembly. crRNA oligos, hammerhead (HH), and hepatitis delta virus (HDV) ribozyme oligos were synthesized by the GeneWiz Co., Ltd., annealed, and subsequently cloned under the U6 promoter within the previously mentioned mammalian expression vector. The sequences mentioned above are listed in Supplementary Tables S1–S6.

### Cell culture and transfection

HEK293T cells, NIH-3T3 cells, sheep fibroblasts, and tdTomato sheep fibroblasts were cultured at 37°C with 5% CO_2_ with high-glucose Dulbecco’s Modified Eagle’s Medium (DMEM, Gibco), 10% (v/v) fetal bovine serum (FBS, Gibco), 1% (v/v) penicillin–streptomycin (100×, Gibco). For transient transfection of HEK293T cells, the cells were seeded in 12-well plates and transfected at ∼70% confluency. 1.5 μg of vectors were transfected into HEK293T cells using Lipofectamine 3000 (Thermo Fisher Scientific) following the manufacturer’s protocol. For transient transfection of NIH-3T3 cells, sheep fibroblasts, and tdTomato sheep fibroblasts, cells were seeded at 70%–80% confluency in 10-cm dishes and then 2 × 10^6^ cells were electroporated with 7 μg of plasmid using Nucleofector II/2b device (Lonza) with program A-033.

### Isolation of primary fibroblasts

A small sample of ear tissue was harvested and disinfected in 75% ethanol for 1 min, washed three times with phosphate-buffered saline, and then minced into ∼1 mm³ pieces with sterile scissors. The tissue was then placed in 200 μl of FBS in a 10-cm dish and incubated upside down at 37°C with 5% CO_2_ for 1 h. After incubation, complete culture medium [DMEM (Gibco), 10% (v/v) FBS (Gibco), and 1% (v/v) penicillin–streptomycin (Gibco)] was gently added to avoid displacing the tissue. Fibroblasts began migrating from the explants after approximately one week. At ∼90% confluency, cells were trypsinized and collected for subsequent transfection.

### Construction of tdTomato reporter cell line

To enable sensitive and efficient detection of editing efficiency across optimization steps, a tdTomato expression cassette was inserted into the single-copy ZFY gene to establish a stably transfected reporter cell line. Using CRISPR–Cas9 combined with homology-mediated end joining (HMEJ), the first intron of the ZFY gene in sheep fibroblasts was targeted to insert the CBh-tdTomato-SV40 poly(A) cassette. This reporter cell line enabled the assessment of CRISPR–Cas-mediated genome editing activity by measuring the ratio of quenched tdTomato fluorescence or weakly fluorescent cells following crRNA targeting.

### Evaluation of gene editing efficiency

A fluorescence reporter system combined with fluorescence-activated cell sorting (FACS) analysis was employed to evaluate editing activity under various conditions, including crRNA scaffold optimization, T5E fusion, codon optimization, and the screening of high-efficiency mutation combinations. Cells were dissociated with 0.25% trypsin-EDTA (Gibco) and suspended in FBS-containing DMEM. To assess Cas12i.3 nuclease activity using the fluorescence reporter system, ∼10 000 single cells were recorded with a BD LSRFortessa 48 h post-transfection. Editing efficiency was determined by measuring the ratio of quenched tdTomato fluorescence and weakly fluorescent cells among EGFP-positive cells.

T7 endonuclease I (T7E1) assays were performed to evaluate editing activity under various conditions, including crRNA scaffold optimization, T5 exonuclease fusion, codon optimization, and single-point mutant screening, as well as for comparisons between IOCas12i and Cas12i.3 or SpCas9. The polymerase chain reaction (PCR) products generated with the PrimeSTAR Max DNA Polymerase were hybridized in NEBuffer 2 (New England Biolabs) on a T100 thermal cycler (Bio-Rad). The primers for amplifying target loci in the T7E1 assay are provided in Supplementary Tables S2, S3, and S6. The annealed samples were then digested with T7E1 (New England Biolabs) for 15 min and separated by electrophoresis on a 2% agarose gel stained with GelStain (TransGen Biotech). The mutation frequency, represented as the percentage of insertions and deletions (indels), was calculated with the formula: Indel (%) = 100 × [1 − (1 − fraction cleaved)^1/2^].

To compare the editing efficiency of optimized constructs (EOCas12i–Combo1 and EOCas12i–Combo2) with Cas12i.3, IOCas12i, CasSF01, SpCas9, and LbCas12a, targeted deep sequencing was performed. Genomic regions of interest were amplified by PrimeSTAR Max DNA Polymerase (Takara). Primers for PCR amplification of target genomic sites are listed in Supplementary Tables S4 and S5. The amplified products were purified using TaKaRa MiniBEST DNA Fragment Purification Kit Ver.4.0 (Takara) and sequenced by a DNBSEQ-T7 platform with 150-bp paired-end reads (Benagen Co., Ltd). Sequencing reads were analyzed by CRISPResso2 [15] for the quantification of editing efficiency.

To evaluate the multiplex editing capability of the EOCas12i platform, whole-genome sequencing (WGS) was performed at 100× coverage using the DNBSEQ-T7 platform. Raw reads were initially quality-filtered and trimmed to ensure a Q score of 30 or higher. The BWA-MEM aligner was used to map the reads to the GRCh38.p14 reference genome, and the resulting BAM files were sorted with Samtools and deduplicated using GATK. The final BAM files were then used to analyze editing efficiencies at each target site with CRISPRessoWGS [15], applying the parameters -wc -4 -w 6.

### Western blot

Cells were lysed in radioimmunoprecipitation assay (RIPA) buffer (Aidlab) supplemented with protease inhibitor cocktail. The supernatants of the lysates were quantified using a BCA Protein Assay Kit (Aidlab) and adjusted to a uniform concentration using RIPA buffer. After heating at 95°C for 5 min, equal volumes of cell lysate were run on 6% polyacrylamide gels and transferred to polyvinylidene difluoride (PVDF) membranes (Millipore). The membrane was then blocked in 5% nonfat milk in Tris-buffered saline with Tween 20 (TBST) buffer and incubated overnight with the primary antibody [anti-DYKDDDDK tag (Beyotime) and anti-Tubulin (Beyotime)] at 4°C. After three washes with TBST, the membranes were incubated with the secondary anti-mouse antibody (ZSGB-BIO) at room temperature for 1 h. The protein band was visualized using chemiluminescent substrates (Invitrogen, WP20005), according to the manufacturer’s instructions.

### Structure-based design of Cas12i.3 variants

The structural model of Cas12i.3 was constructed using the SWISS-MODEL server (https://swissmodel.expasy.org), based on the structure of Cas12i2. The associated crRNA scaffold was similarly adapted from that of Cas12i2, substituting the sequence with validated ZFX and EMX1 target sites [16]. Cas12i.3 was then paired with crRNAs targeting ZFX or EMX1, and PyMOL (Version 2.3.2) was used to identify Cas12i.3 residues within 2 Å of the crRNAs targeting ZFX or EMX1. In parallel, Cas12i.3–crRNA complexes for ZFX or EMX1 were analyzed with protein–ligand interaction profiler (PLIP, https://plip-tool.biotec.tu-dresden.de) to identify interacting residues. Structural alignment with Cas12i2 further revealed three evolutionarily conserved residues in Cas12i.3—K181, Q432, and T453—which correspond to positions previously shown to enhance editing efficiency when mutated in Cas12i2 [17]. These conserved residues were therefore also included in the mutational screen. All candidate residues identified from spatial proximity, interaction profiling, and conserved functional sites were pooled for mutagenesis. Among these, non-positively charged residues were mutated to arginine to generate Cas12i.3 variants with potentially enhanced editing activity.

### Generation of crRNA and mRNA from *in vitro* transcription

To generate linear DNA templates for *in vitro* transcription (IVT), IOCas12i and EOCas12i coding region with T7 promoter was amplified by PCR using Q5 High-Fidelity DNA Polymerase (New England Biolabs) and primers IVT-mRNA-F and -R (IVT-mRNA-F: 5′-TAATACGACTCACTATAGGGGCCACCATGGACTATAAGGACC-3′ and IVT-mRNA-R: 5′-TCCCCAGCATGCCTGCTATTCT-3′). The PCR products were purified using the TaKaRa MiniBEST DNA Fragment Purification Kit Ver.4.0 (Takara) and eluted in nuclease-free water. IVT reactions were performed using mMESSAGE mMACHINETM T7 ULTRA Kit (Ambion), following the manufacturer’s instructions. The resulting mRNA was eluted in nuclease-free water and stored at −80°C until use.

For crRNA synthesis, crRNA targeting DNMT1 locus with T7 promoter was amplified by PCR using the same polymerase but with primer IVT-DNMT1-F and -R (IVT-DNMT1-F: 5′-TAATACGACTCACTATAGGGAGAGAATGTGCGCATAGTCGCAC-3′ and IVT-DNMT1-R: 5′-AGGTCATCTACAAAGCCCCTTCTGTGCGACTATGCGCACA-3′). The PCR products were purified using the TaKaRa MiniBEST DNA Fragment Purification Kit Ver.4.0 (Takara) and eluted in nuclease-free water. IVT reactions for crRNA were performed using the MEGAshortscript^™^ T7 kit (Ambion), following the manufacturer’s instructions. The resulting crRNA was eluted in nuclease-free water and stored at −80°C until use.

### Animals and microinjection of zygotes

Seven- to eight-week-old female mice were injected with 5 IU of pregnant mare serum gonadotropin, followed 48 h later by 5 IU of human chorionic gonadotropin. Hormone-treated females were then placed in a mating with fertile stud males and embryos were collected the following day.

For crRNA and mRNA delivery, mixed crRNA and mRNAs were microinjected into zygotes from female C57 mice. After microinjections, embryos were either immediately transferred into pseudopregnant females or cultured in KSOM until they reached the blastocyst stage (E4.5). To generate post-implantation embryos or live pups, microinjected embryos were surgically transferred into the oviducts of pseudopregnant females immediately after microinjection. Ear clips were collected from 2-week-old mice, and genomic DNA was extracted using HiPure Tissue DNA Micro Kit (Magen Biotech). PCR was conducted on extracted DNA with PrimeSTAR Max DNA Polymerase (Takara).

Individual blastocyst stage embryos were incubated in 10 μl of embryo lysis buffer at 55°C for 30 min. PCR was performed using a 1 μl of sample with PrimeSTAR Max DNA Polymerase (Takara). The PCR products from the edited embryos or mice (ear clip samples) were subjected to 12% polyacrylamide gel electrophoresis, stained with GelStain (TransGen Biotech) for 30 min, and visualized using a UV transilluminator (Azure Biosystems). The primers used are listed in the Supplementary Table S2.

### Amplicon-based off-target analysis

Forty-eight hours post-transfection, EGFP-positive cells (20 000–40 000 cells) were collected and genomic DNA was extracted using HiPure Tissue DNA Micro Kit (Magen Biotech). Potential off-target sites were predicted by Cas-OFFinder (https://www.rgenome.net/cas-offinder/, with mismatch numbers ≤4 and DNA bulge size ≤2) and amplified by PCR with PrimeSTAR Max DNA Polymerase (Takara). Off-target site information and primers were listed in Supplementary Table S4. The amplicons were purified using TaKaRa MiniBEST DNA Fragment Purification Kit Ver.4.0 (Takara) and sequenced by a DNBSEQ-T7 platform with 150-bp paired-end reads (Benagen Co., Ltd). Sequencing reads were analyzed by CRISPResso2 for the quantification of editing efficiency.

### GUIDE-seq analysis

GUIDE-seq was employed to assess the on-target and off-target frequencies of EOCas12i–Combo1, EOCas12i–Combo2, and SpCas9 targeting ADRB2 and CXCR4, as previously described [18, 19]. GUIDE-seq and data analysis were completed by GeneRulor Co., Ltd. Briefly, 5 μl of dsODN (100 μM) and 10 μg of plasmids expressing nucleases and guide RNAs (gRNAs) were co-transfected into HEK293T cells. After 72 h, DNA was extracted and subjected to dsODN–PCR for cleavage verification. GUIDE-seq libraries were generated by DNA fragmentation, Y-adapter ligation, two rounds of PCR, and sequencing on the MGISEQ-2000RS (PE150, paired-end) with customized settings for 16-bp UMIs. The resulting data were demultiplexed using in-house Python scripts and analyzed using GUIDEseq v1.1 [20].

## Results

### Scaffold RNA engineering in CRISPR–Cas12i.3

**Figure 1.**
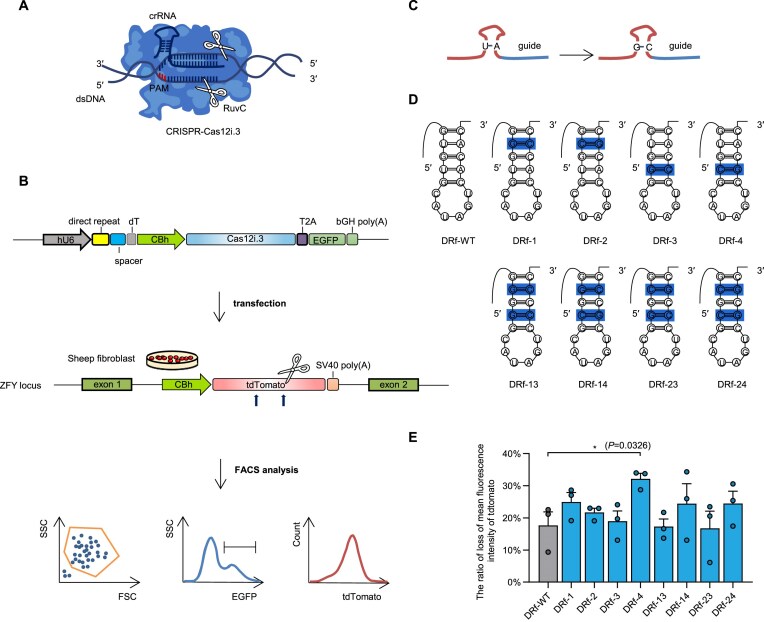
Stem–loop engineering of Cas12i.3 crRNA enhances editing efficiency in sheep fibroblasts. (**A**) Schematic of the Cas12i.3–crRNA–dsDNA ternary complex. PAM, protospacer-adjacent motif. dsDNA, double-stranded DNA. Scissors denote the cleavage sites. (**B**) The workflow for assessing Cas12i.3 editing activity via tdTomato fluorescence in sheep fibroblasts by flow cytometry. A plasmid encoding Cas12i.3, EGFP, and crRNA was constructed, along with a sheep fibroblast cell line with tdTomato integrated at the ZFY locus. (**C**) Stem–loop engineering of crRNAs. Maintaining the original base pairing within the stem–loop while moving from an A:U pairing to a G:C pairing to increase the stability of the stem–loop structure. (**D**) Stem–loop engineering of crRNAs. Nucleotides marked indicate sequence differences from the wild-type. DRf-WT, wild-type DRf-crRNA. (**E**) Disruption of tdTomato using Cas12i.3 and stem–loop-engineered crRNAs in sheep fibroblasts (*n* = 3 samples). Mean ± SE, *P < 0.05 (unpaired Student’s *t*-test).

Cas12i.3 is a single crRNA-guided endonuclease with small protein size and simple PAM requirements, which recently discovered CRISPR–Cas system from metagenomes (Fig. 1A and Supplementary Table S1). We first tested genome-editing of this naturally occurring Cas12i.3 in mammalian cells. A construct expressing Cas12i.3 and a ZFX-targeting crRNA was generated (Supplementary Fig. S1A). SpCas9 targeting the same ZFX site served as a reference. The indel frequency of Cas12i.3 was only 8.6% in sheep fibroblasts, whereas that of SpCas9 reached 15.2%, nearly double the efficiency of Cas12i.3 (Supplementary Fig. S1B–D and Supplementary Table S2).

To expand the utility of this compact protein, we aimed to systematically optimize CRISPR–Cas12i.3. We first developed a tdTomato fluorescent reporter cell line based on sheep fibroblasts for evaluating editing efficiency during optimization (Supplementary Fig. S2). In this system, crRNA-guided cleavage within the tdTomato coding sequence results in frameshift mutations that either lead to nonfunctional proteins or introduce premature stop codons resulting in truncation, as well as in-frame deletions that remove essential residues—both of which lead to fluorescence quenching. The reporter cell line was generated by integrating tdTomato into the endogenous single-copy ZFY locus, enabling sensitive assessment of genome-editing efficiency by measuring the ratio of quenched fluorescence and weakly fluorescent cells following transfection with Cas12i.3 and tdTomato-targeting crRNA (Fig. 1B).

In the native CRISPR system, full-length Direct-Repeat crRNA (DRf-crRNA) is initially expressed and then processed by Cas12a into truncated Direct-Repeat crRNA (DRt-crRNA) [21]. In Cas12a, DRf-crRNA is more efficient than DRt-crRNA [10]. Additionally, extending the 5′ end of crRNA in Cas12a improves the editing efficiency [22]. To examine the impact of DRf-crRNA and DRt-crRNA on the nuclease activity of Cas12i.3, we transfected the tdTomato fluorescent reporter cells with the plasmids expressing Cas12i.3 and either DRf-crRNA or DRt-crRNA targeting tdTomato. The quenched fluorescence ratio of DRt-crRNA was 1.8-fold that of DRf-crRNA, with a similar trend observed for weakly fluorescent cells (Supplementary Fig. S3A, C, and E, and Supplementary Tables S1 and S2). T7E1 assay at the endogenous ZFX locus showed that DRt-crRNA achieved 1.6-fold the efficiency of DRf-crRNA in sheep fibroblasts (Supplementary Fig. S3H, and Supplementary Tables S1 and S2). These data indicate that DRt-crRNA is more effective than DRf-crRNA in the context of Cas12.3.

RNA polymerase III (Pol III) promoters preferentially initiate transcription with a guanine (G), but DRt-crRNA lacks a 5′ G, necessitating an extra nucleotide that may affect its activity. Moreover, Pol III-transcribed crRNAs often carry variable 3′ U-tails, which can influence editing efficiency [23]. To express precise DRt-crRNA efficiently, we inserted self-cleaving ribozymes—the HH and HDV ribozymes—at the 5′ and/or 3′ ends of the crRNA. Three crRNA expression plasmids were tested (HH–crRNA, crRNA–HDV, and HH–crRNA–HDV), all driven by the U6 promoter. For clarity, these are referred to as HH, HDV, and HH–HDV, respectively. Flow cytometry revealed that all three constructs exhibited significantly lower ratios of quenched fluorescence and weakly fluorescent cells compared to the DRt-crRNA control (Supplementary Fig. S3B, D, and F, and Supplementary Tables S1 and S2). Similar results were observed across the groups at the sheep endogenous ZFX locus (Supplementary Fig. S3I, and Supplementary Tables S1 and S2). These results indicate that HH and/or HDV ribozyme did not enhance CRISPR–Cas12i.3 editing efficiency.

Mismatched base pairs in the crRNA stem–loop impair Cas12a activity, while A:U to G:C substitutions has been shown to improve editng efficiency by stabilizing crRNA [10, 24]. To explore whether similar stem–loop engineering could enhance Cas12i.3 function, eight stem–loop modifications were engineered by replacing A:U pairs with G:C pairs and were named DRf-1 to DRf-8 (Fig. 1C and D, and Supplementary Table S1). Among them, DRf-4 exhibited the highest activity, with a quenched fluorescence ratio of 32.2%, which was 1.8-fold that of wild-type DRf-crRNA (17.7%; DRf-WT; Fig. 1E, and Supplementary Tables S1 and S2). Additionally, DRf-4 significantly increased the ratio of weakly fluorescent cells from 7.9% in DRf-WT to 10.7% (Supplementary Fig. S3G, and Supplementary Tables S1 and S2). At the sheep endogenous ZFX locus, DRf-4 similarly demonstrated superior editing efficiency (Supplementary Fig. S3J, and Supplementary Tables S1 and S2). These results establish DRf-4 as a structurally optimized crRNA that significantly improves Cas12i.3-mediated genome editing.

### Exonuclease fusion and codon optimization in CRISPR–Cas12i.3

**Figure 2.**
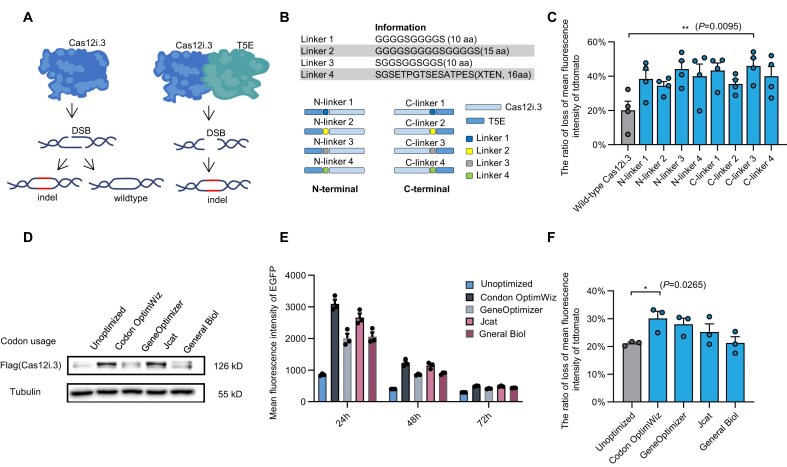
Effects of Cas12i.3 codon optimization and fusion of Cas12i.3 with exonuclease on gene editing of CRISPR–Cas12i.3 in sheep fibroblasts. (**A**) Schematic of the different DNA repair outcomes favored by the CRISPR–Cas12i.3 system with/without T5 exonuclease (T5E) fusion. DSB: double-stranded breaks; indel: insertion and deletion. (**B**) Schematic of T5E fused to the N- or C-terminus of Cas12i.3 via different linkers. (**C**) Disruption of tdTomato by Cas12i.3 fused with T5E at either the N- or C-terminus via different linkers in sheep fibroblasts (*n* = 4 samples). “N-linker 1” refers to the fusion of T5E to the N-terminal of Cas12i.3 using linker 1, with similar meanings for “N-linker 2,” “N-linker 3,” and so on. (**D**) Western blot showing expression levels of five codon-optimized Cas12i.3 constructs. (**E**) EGFP expression of five codon-optimized Cas12i.3 after transfection at 24, 48, and 72 h in sheep fibroblasts (*n* = 3 samples). (**F**) Effects of five different codon usages on cleavage efficiency of Cas12i.3 in sheep fibroblasts (*n* = 3 samples). The unoptimized condition represents sequences that have not undergone mammalian codon optimization. Mean ± SE, **P < 0.01; *P < 0.05 (unpaired Student’s *t*-test).

Given that Cas12i.3 generates DNA cleavage products with long 5′ overhangs prone to accurate repair without causing indels, we investigated whether a 5′ exonuclease could enhance its editing efficiency by removing these overhangs (Fig. 2A). T5E has previously been shown to boost editing efficiency for Cas9 and Cas12a [25, 26]. We fused T5E to either the N- or C-terminus of the Cas12i.3 protein using four distinct linkers: (GGGGS)_2_, (GGGGS)_3_, SGGSGGSGGS, and XTEN, designated as linker 1, linker 2, linker 3, and linker 4, respectively. The term “N-linker 1″ refers to the fusion of T5E to the N-terminus of Cas12i.3 using linker 1, with similar meanings for “N-linker 2,” “N-linker 3,” and so on (Fig. 2B and Supplementary Table S1). Flow cytometry revealed that C-linker 3 achieved the highest quenched fluorescence ratio (46.1%), which was 2.3-fold that of the control (20.0%) (Fig. 2C and Supplementary Table S2). Furthermore, C-linker 3 significantly increased the proportion of weakly fluorescent cells within the EGFP-positive population compared to the control (8.4% versus 5.0%; Supplementary Fig. S4A and Supplementary Table S2). Consistent with these findings, T7E1 assays on the endogenous ZFX locus in sheep fibroblasts demonstrated that both C-linker 1 and C-linker 3 yielded an editing efficiency of 15.9%, a 35.9% improvement over the control (11.7%; Supplementary Fig. S4B and Supplementary Table S2). Taken together, these results indicate that the fusion of T5E to the C-terminus of Cas12i.3 via linker 3 (amino acid sequence: SGGSGGSGGS) most effectively enhances Cas12i.3-mediated genome editing.

Codon optimization of Cas12i.3 was further explored as a strategy to enhance editing efficiency by improving protein expression through alignment with the host’s codon usage. We evaluated four mammalian-optimized codon schemes (Codon OptimWiz, GeneOptimizer, Jcat, and General Biol) alongside an unoptimized sequence. All optimized schemes showed increased codon adaptation index (CAI) values for both humans and sheep, with Codon OptimWiz and Jcat group achieving near-maximal CAI values (Supplementary Fig. S4C and Supplementary Table S1). However, Jcat had >70% GC content in certain regions, potentially affecting expression (Supplementary Fig. S4D). Fluorescence microscopy, flow cytometry, and western blotting confirmed Codon OptimWiz as the top performer in Cas12i.3 expression (Fig. 2D and E, and Supplementary Fig. S4E).

To further investigate the four codon optimizations on Cas12i.3 editing efficiency, expression vectors with different codon optimizations were constructed. Quenched fluorescence ratio for the Unoptimized, Codon OptimWiz, GeneOptimizer, Jcat, and General Biol groups were 21.1%, 30.1%, 28.0%, 25.2%, and 21.3%, respectively, with Codon OptimWiz group achieving the highest rate at 1.4-fold that of the Unoptimized group (Fig. 2F and Supplementary Table S2). Similarly, a higher proportion of weakly fluorescent cells was observed in the Codon OptimWiz group compared to the Unoptimized group (Supplementary Fig. S4F and Supplementary Table S2). For the endogenous ZFX locus, Codon OptimWiz, GeneOptimizer, and Jcat groups also showed higher editing efficiencies, at 15.6%, 15.8%, and 14.5%, respectively, while Unoptimized and General Biol groups showed lower efficiencies of 13.2% and 8.2%, respectively (Supplementary Fig. S4G and Supplementary Table S2). Together, these data demonstrate that Codon OptimWiz optimizations enhance both Cas12i.3 expression and editing efficiency.

### Integration of crRNA engineering and codon optimization in IOCas12i development

**Figure 3.**
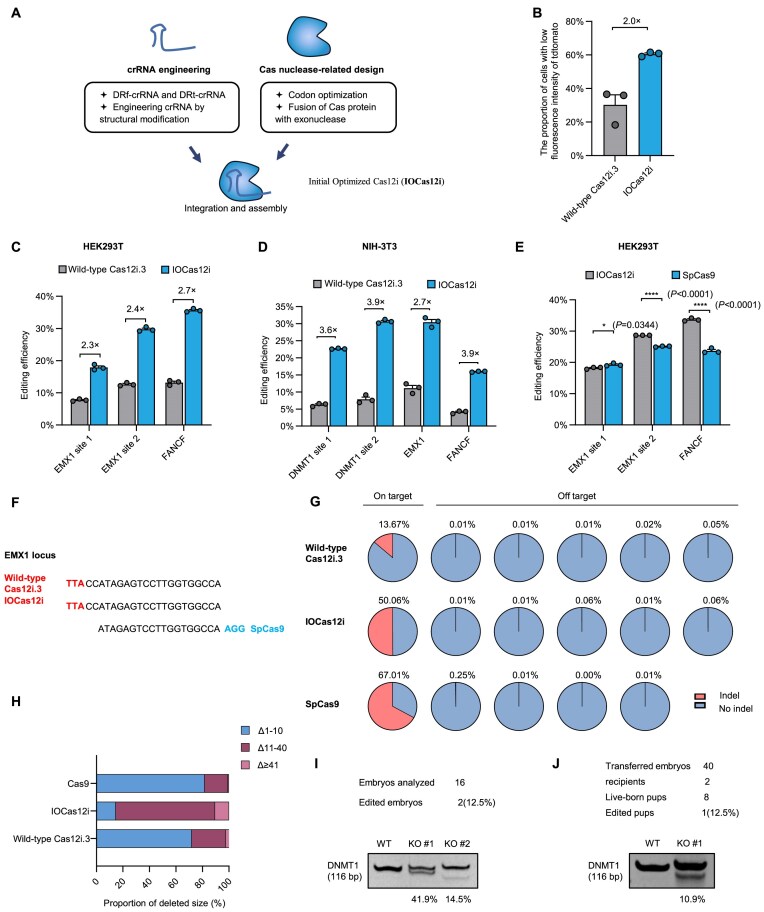
Boosting CRISPR–Cas12i.3 genome editing capability through combining two comprehensive optimization steps. (**A**) The combination of engineered crRNA, Cas12i.3 codon optimization, and fusion with T5 exonuclease produces a highly efficient IOCas12i system. (**B**) IOCas12i-mediated gene-editing efficiency for the tdTomato in sheep fibroblasts (*n* = 3 samples). (**C**) Indel efficiencies by Cas12i.3 and IOCas12i at three endogenous target loci in HEK293T cells (*n* = 3 samples), determined by T7E1 assay. Gel images are shown in Supplementary Fig. S5. (**D**) Indel efficiencies by Cas12i.3 and IOCas12i at four endogenous target loci in NIH-3T3 cells (*n* = 3 samples), determined by T7E1 assay. Gel images are shown in Supplementary Fig. S5. (**E**) Indel efficiencies by IOCas12i and SpCas9 at three endogenous target loci in HEK293T cells (*n* = 3 samples), determined by T7E1 assay. Gel images are shown in Supplementary Fig. S5. (**F**) Sequence of the human EMX1 gene protospacer that is targeted by Cas12i.3, IOCas12i, and SpCas9. (**G**) The indel percentages at on-target and predicted off-target sites analyzed by targeted deep sequencing. (**H**) Indel patterns generated by Cas12i.3, IOCas12i, and SpCas9 at EMX1 locus in HEK293T cells, determined by targeted deep sequencing. (**I**) IOCas12i-mediated editing at the DNMT1 locus in ICR mouse embryos, determined by PAGE electrophoresis. WT, wild-type mouse embryo; KO, DNMT1-knockout mouse embryo. (**J**) IOCas12i-mediated editing at the DNMT1 locus in the edited pup, determined by PAGE electrophoresis; WT, wild-type mouse; KO, DNMT1-knockout mouse. Mean ± SE, ****P < 0.0001; *P < 0.05 (unpaired Student’s *t*-test).

To further enhance efficiency, we integrated optimized crRNA design, Cas12i.3 codon optimization, and exonuclease fusion to develop IOCas12i system (Fig. 3A). Interestingly, a C-to-A mutation at the third codon of amino acid 273 (S273R) in IOCas12i occurred by chance during vector construction. Given IOCas12i’s superior editing efficiency, this mutation was retained. Compared with Cas12i.3, IOCas12i exhibited a quenched fluorescence ratio 2.0-fold that of Cas12i.3, with a similar trend observed for weakly fluorescent cells (Fig. 3B, and Supplementary Fig. S5A and Supplementary Table S2). Editing efficiency at the endogenous ZFX locus increased from 11.2% with Cas12i.3 to 26.8% with the optimized IOCas12i, as measured by T7E1 assay (Supplementary Fig. S5B and Supplementary Table S2). To evaluate the broad applicability of IOCas12i across mammalian species, we next assessed its editing performance in human HEK293T and mouse NIH-3T3 cells. In HEK293T cells, IOCas12i achieved 2.3–2.7-fold the editing efficiencies of Cas12i.3 at EMX1 site 1, EMX1 site 2, and FANCF (Fig. 3C, and Supplementary Fig. S5C–E and Supplementary Table S3). In NIH-3T3 cells, IOCas12i demonstrated 2.7–3.9-fold the editing efficiencies of Cas12i.3 at DNMT1 site 1, DNMT1 site 2, EMX1, and FANCF (Fig. 3D, and Supplementary Fig. S5F–I and Supplementary Table S3). Across all tested sites, Cas12i.3 and IOCas12i showed average editing efficiencies of 11.2% and 27.8%, respectively, in HEK293T cells, and 7.3% and 24.9% in NIH-3T3 cells. We further compared the editing activity of IOCas12i and SpCas9 at EMX1 site 1, EMX1 site 2, and FANCF in HEK293T cells using identical spacer sequences (5′-TTN PAM for IOCas12i and NGG-3′ PAM for SpCas9). At EMX1 site 1, SpCas9 was 1.1 times as efficient as IOCas12i. In contrast, at EMX1 site 2 and FANCF, IOCas12i achieved 1.1 and 1.4 times the efficiency of SpCas9, respectively (Fig. 3E, and Supplementary Fig. S5J–L and Supplementary Table S3). Importantly, both Cas12i.3 and IOCas12i exhibited minimal off-target activity, whereas SpCas9 showed potential off-target effects at a predicted site (Fig. 3F and G, and Supplementary Table S4). These findings demonstrate that IOCas12i, through the integration of optimized crRNA, codon-optimized Cas12i.3, and exonuclease fusion, substantially enhances genome-editing efficiency without increasing off-target effects.

To evaluate IOCas12i’s impact on deletion length, PCR amplicons surrounding the gRNA target sites were subjected to next-generation sequencing (NGS) analysis. The results revealed that IOCas12i primarily produces deletions of 11–40 bp (74.8%), whereas Cas12i.3 and SpCas9 predominantly generated shorter deletions of 0–10 bp (71.9% and 81.7%, respectively), as shown in Fig. 3H, and Supplementary Fig. S6 and Supplementary Table S4. Overall, these findings indicate that IOCas12i induces longer deletions compared to Cas12i.3 and SpCas9.

To assess the applicability of IOCas12i for embryo editing and gene-edited animal generation, we employed a triplex structure to place the crRNA targeting the mouse DNMT1 gene downstream of the IOCas12i coding sequence. *In vitro*-transcribed mRNA was microinjected into single-cell mouse embryos, which were subsequently cultured to the blastocyst stage and lysed for editing efficiency analysis via polyacrylamide gel electrophoresis (PAGE). Among 16 embryos analyzed, two displayed additional shorter bands. TA cloning and sequencing revealed 9- and 18-bp deletions in these embryos, indicating that 2 out of 16 embryos (12.5%) carried detectable edits (Fig. 3I, and Supplementary Fig. S7A and Supplementary Table S2). Subsequently, 40 embryos were transferred into two recipient females, resulting in eight pups. One pup exhibited marked growth retardation and appeared noticeably smaller than its littermates. PAGE and sequencing revealed an 18-bp deletion at the DNMT1 locus in this individual, representing an editing efficiency of 12.5% (1/8) among the offspring (Fig. 3J, Supplementary Fig. S7B and C; Supplementary Table S2). These findings demonstrate the efficacy of IOCas12i for both embryo editing and gene-edited animal generation.

### Combinatorial mutagenesis of IOCas12i across functional domains

**Figure 4.**
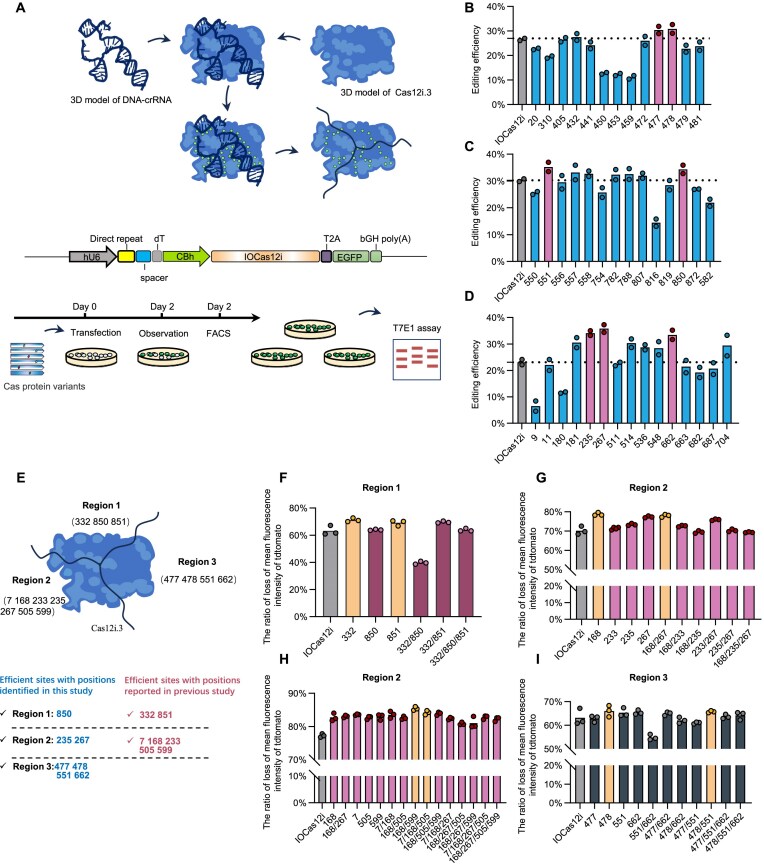
Identification of highly effective mutation combinations within three regions of IOCas12i. (**A**) Rationale and workflow for the construction of single-mutation IOCas12i variants. Potential mutation sites in Cas12i.3 were identified through the analysis of its 3D-reconstructed ternary complex with crRNA and DNA. Residues in Cas12i.3 that interact with the crRNA–DNA complex, as well as those within 2 Å of the crRNA–DNA binary complex, were grouped into three regions based on their positions in IOCas12i. Structural alignment with Cas12i2 further revealed three evolutionarily conserved residues in Cas12i.3—K181, Q432, and T453—which correspond to positions previously shown to enhance editing efficiency when mutated in Cas12i2. These residues, totaling 42, were mutated to arginine, and their editing efficiencies were compared within each region to identify the most effective sites. Ultimately, these sites will be used for further combinations to enhance editing efficiency. (**B–** **D**) The editing efficiency of single-mutation IOCas12i variants in sheep fibroblasts, determined by T7E1 assay (*n* = 2 samples). Gel images are shown in Supplementary Fig. S8. Numbers (e.g. 20 and 310) represent the positions of amino acid residues in the protein sequence that are mutated to arginine (R). (**E**) Rationale and workflow for the construction of highly efficient IOCas12i variants with multiple mutations. The efficient sites identified in Fig. 4B–D, along with those reported in the literature, were compiled and grouped into three regions based on their positions within IOCas12i: Region 1, Region 2, and Region 3. The sites within each region were then combined, and their cleavage activities were assessed using the method described in Fig. 1B. For Region 2, a two-step process was necessary due to its higher site density. Finally, the highly efficient sites from all three regions were used to assemble the final variant. (**F–I**) Disruption of tdTomato by different mutation combinations in IOCas12i regions 1 (F), 2 (G and H), and 3 (I), as determined by flow cytometry in sheep fibroblasts (*n* = 3 samples). Numbers (e.g. 332 and 850) represent the positions of amino acid residues in the protein sequence that are mutated to arginine (R); mean ± SE.

To further enhance genome-editing activity of IOCas12i, we applied a protein engineering strategy targeting its DNA–crRNA binding pocket. We hypothesized that certain amino acid substitutions with this region might modulate the interaction between the Cas protein and DNA or crRNA. To test this, we introduced a total of 42 individual amino acid substitutions at three distinct positions within the binding interface and constructed a plasmid library co-expressing each IOCas12i variant with EGFP. Following transfection of HEK293T cells and FACS sorting of EGFP-positive populations, genomic DNA was extracted and editing activity was assessed using T7E1 assays (Fig. 4A and Supplementary Fig. S8A). Several mutations were found to significantly improve editing efficiency in mammalian cells, including T235R, D267R, S477R, G478R, D551R, L662R, and T850R. In the next stage, we combined these mutations with a set of previously reported beneficial substitutions, including S7R, N168R, D233R, L332R, T505R, S599R, and D851R (Fig. 4B–D and Supplementary Fig. S8B–G). To systematically evaluate the combinatorial effect of these mutations, we grouped them based on their spatial proximity into three regions—designated Region 1, Region 2, and Region 3. Within each region, representative combinations were constructed and evaluated using a tdTomato-targeting assay in fluorescent reporter sheep fibroblasts (Fig. 4E–I and Supplementary Fig. S9). In Region 1, among the six tested single and combined variants, L332R and D851R individually conferred the greatest enhancement in editing efficiency, each reaching 1.1-fold that of the IOCas12i (Fig. 4F and Supplementary Fig. S9A). In Region 2, due to the higher density of beneficial candidates, we initially evaluated various combinations of N168R, D233R, T235R, and D267R. Among the eight tested variants, N168R and N168R/D267R showed the most notable improvement. Further gains were achieved by incorporating S7R, T505R, and S599R into these backbones, with N168R/S599R and S7R/N168R/T505R emerging as top performers (Fig. 4G and H, and Supplementary Fig. S9B and C). In Region 3, of 11 variants tested, G478R and G478R/D551R demonstrated the most pronounced enhancement in editing activity (Fig. 4I and Supplementary Fig. S9D).

### Generating highly efficient systems EOCas12i by combining multiple optimizations

Having identified the most effective amino acid substitutions in each of the three defined structural regions of IOCas12i, we next sought to integrate these region-specific mutations to generate enhanced variants. Because IOCas12i itself incorporates multiple layers of optimization—including engineered crRNA architecture, codon usage adaptation, and exonuclease fusion—the resulting variants inherently combine these features with the newly introduced amino acid substitutions.

**Figure 5.**
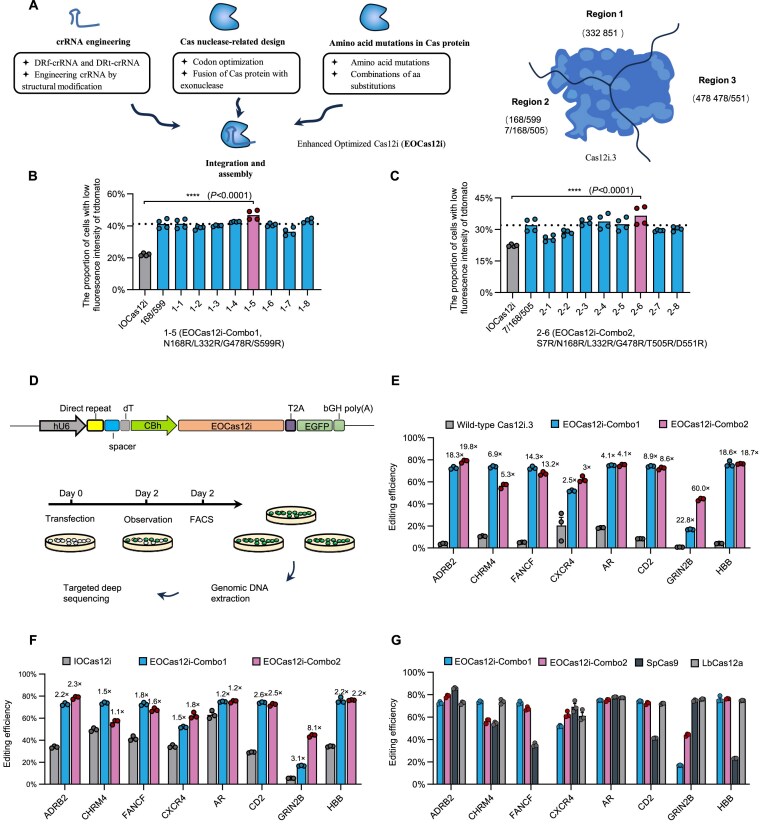
Advancing the genome editing efficiency through assembling multiple optimization steps. (**A**) The integration of engineered crRNA, Cas12i.3 codon optimization, fusion of exonuclease, amino acid mutations, and their strategic combination results in highly efficient EOCas12i systems. In essence, we further integrate the highly effective mutations identified within the three regions of IOCas12i to generate the final optimized variant, EOCas12i. Numbers (e.g. 332 and 850) represent the positions of amino acid residues in the protein sequence that are mutated to arginine (R). (**B** and **C**) Further combinations of mutation combinations identified from the three-region screens generated variants with even higher editing efficiency, determined using the method described in Fig. 1B (*n* = 4 samples). Numbers (168/599 and 7/168/505) represent the positions of amino acid residues in the protein sequence that are mutated to arginine (R). (**D**) The experimental workflow for detecting wild-type Cas12i.3, IOCas12i, EOCas12i–Combo1, EOCas12i–Combo2, SpCas9, and LbCas12a cleavage activity in endogenous loci. (**E**) Indel efficiencies by Cas12i.3, EOCas12i–Combo1, and EOCas12i–Combo2 at eight endogenous target loci in HEK293T cells (*n* = 3 samples), determined by targeted deep sequencing. (**F**) Indel efficiencies by IOCas12i, EOCas12i–Combo1, and EOCas12i–Combo2 at eight endogenous target loci in HEK293T cells (*n* = 3 samples), determined by targeted deep sequencing. (**G**) Indel efficiencies by EOCas12i–Combo1, EOCas12i–Combo2, SpCas9, and LbCas12a at eight endogenous target loci in HEK293T cells (*n* = 3 samples), determined by targeted deep sequencing. The same spacer sequences were used, with a 5′-TTN PAM for Cas12i, a 5′-TTTV PAM for LbCas12a, and an NGG-3′ PAM for SpCas9. Mean ± SE, ****P < 0.0001 (unpaired Student’s *t*-test).

Based on prior data, N168R/S599R and S7R/N168R/T505R represented the top-performing combinations in Region 2. Building upon these backbones, we further introduced high-efficiency substitutions from Regions 1 and 3 to generate two series of variants: combinations 1–1 to 1–8 (derived from N168R/S599R) and combinations 2–1 to 2–8 (from S7R/N168R/T505R). Among the N168R/S599R-derived combinations, combinations 1–5 (N168R/L332R/G478R/S599R) exhibited the highest editing efficiency, while combinations 2–6 (S7R/N168R/L332R/G478R/T505R/D551R) showed the best performance in the S7R/N168R/T505R background. The top-performing constructs were designated as EOCas12i–Combo1 and EOCas12i–Combo2, respectively (Fig. 5A–C and Supplementary Fig. S10).

To evaluate the genome-editing capabilities of the optimized variants EOCas12i–Combo1 and EOCas12i–Combo2, we performed deep-targeted sequencing at eight representative endogenous loci—ADRB2, CHRM4, FANCF, CXCR4, AR, CD2, GRIN2B, and HBB—in HEK293T cells. On average, EOCas12i–Combo1 and EOCas12i–Combo2 generated indels at these eight sites with frequencies of 64.1% and 66.6%, respectively. Compared with wild-type Cas12i.3, the two engineered variants exhibited editing efficiencies ranging from 2.5- to 22.8-fold and 3.0- to 60.0-fold those of Cas12i.3 (Fig. 5D and E, and Supplementary Table S5). Relative to IOCas12i, their editing efficiency ranged from 1.2- to 3.1-fold (Combo1) and 1.1- to 8.1-fold (Combo2) those of IOCas12i across the tested sites (Fig. 5F and Supplementary Table S5). Notably, at the CHRM4, FANCF, CD2, and HBB loci, both EOCas12i variants surpassed SpCas9 in editing efficiency, while showing comparable or slightly reduced activity at ADRB2, CXCR4, and AR, with the exception of GRIN2B. When benchmarked against LbCas12a, both EOCas12i–Combo1 and EOCas12i–Combo2 demonstrated similar editing efficiencies at all loci except GRIN2B (Fig. 5G and Supplementary Table S5). A similar trend was observed when compared with CasSF01, with both variants showing comparable editing efficiencies at seven of the eight tested loci (Supplementary Fig. S11). Collectively, these results highlight the strong and competitive editing performance of EOCas12i–Combo1 and EOCas12i–Combo2.

### Functional characterization and applications of EOCas12i

**Figure 6.**
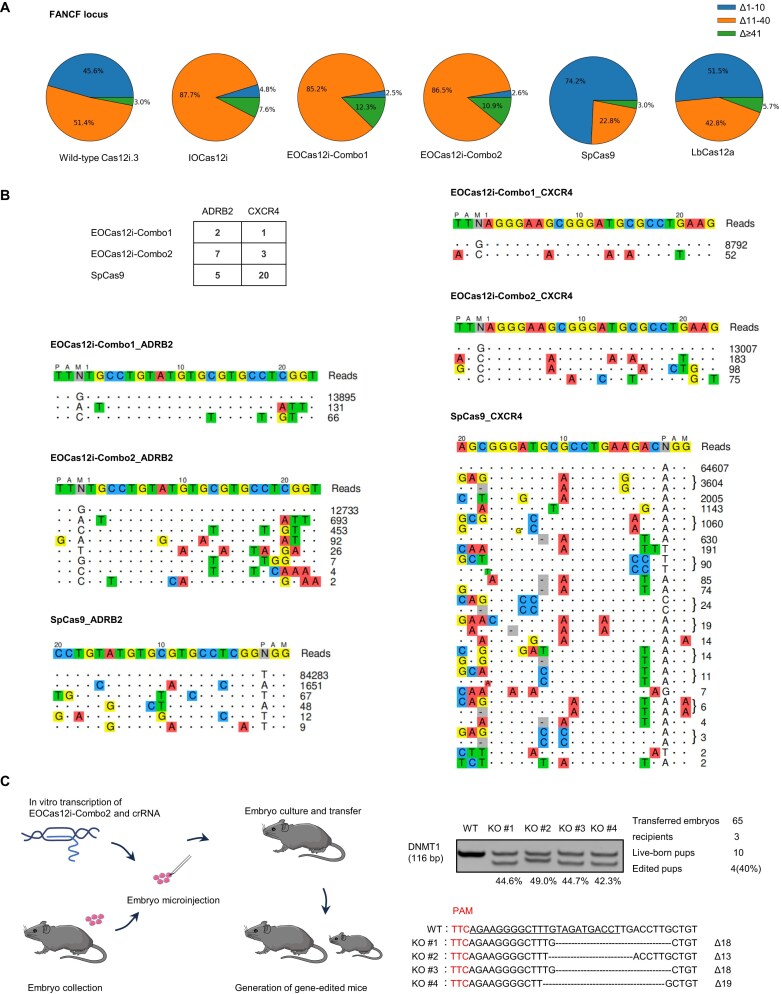
Functional characterization and applications of EOCas12i. (**A**) EOCas12i induces longer genomic deletions in FANCF locus compared to Cas12i.3, IOCas12i, SpCas9, and LbCas12a in HEK293T cells. (**B**) Off-target sites identified by GUIDE-seq for EOCas12i–Combo1, EOCas12i–Combo2, and SpCas9 in HEK293T cells. (**C**) Application of EOCas12i–Combo2 for knockout at the DNMT1 locus in a mouse. Polyacrylamide gel electrophoresis revealed an additional shorter band in the mouse born from an embryo treated with EOCas12i–Combo2 and subsequently transplanted, compared to the wild-type mouse. TA cloning and sequencing further confirmed genomic deletions in these mouse.

To assess EOCas12i’s impact on deletion length, we performed deep sequencing analysis on PCR amplicons encompassing gRNA target sites in HEK293T cells. Both the IOCas12i and EOCas12i systems induced a higher proportion of deletions ranging from 11 to 40 bp at ADRB2, CHRM4, FANCF, and CXCR4, whereas deletions by Cas12i.3, SpCas9, and LbCas12a were predominantly restricted to 10 bp or smaller, especially for SpCas9 (Fig. 6A, and Supplementary S12 and Supplementary Table S5). These findings indicate that EOCas12i shares a similar deletion pattern with IOCas12i, favoring the generation of larger deletions.

To investigate the specificities of EOCas12i, we analyzed the genome-wide off-target effects of EOCas12i–Combo1, EOCas12i–Combo2, and SpCas9 at ADRB2 and CXCR4 loci using GUIDE-seq in HEK293T cells. At the ADRB2 locus, the number of off-target sites was similar for all three systems. In contrast, at the CXCR4 locus, SpCas9 exhibited more off-target sites than both EOCas12i–Combo1 and EOCas12i–Combo2 (Fig. 6B). These results suggest that EOCas12i–Combo1 and EOCas12i–Combo2 may offer superior specificity compared to SpCas9.

To further evaluate the application of the EOCas12i–Combo2 in generating gene-edited animals, *in vitro*-transcribed mRNA and crRNA targeting DNMT1 were microinjected into collected single-cell mouse embryos. A total of 65 two-cell embryos were transferred into the oviducts of three recipient females, resulting in ten pups. PAGE analysis of the genomic DNA from these pups revealed that four (40%) exhibited additional shorter bands alongside the wild-type, indicative of indel mutations. Subsequent TA cloning and sequencing confirmed deletions at the DNMT1 locus (Fig. 6C and Supplementary Table S2). These results validate the effectiveness of the EOCas12i–Combo2 system in generating gene-edited animals.

### Developing EOCas12i-based multiplexed genome editing

**Figure 7.**
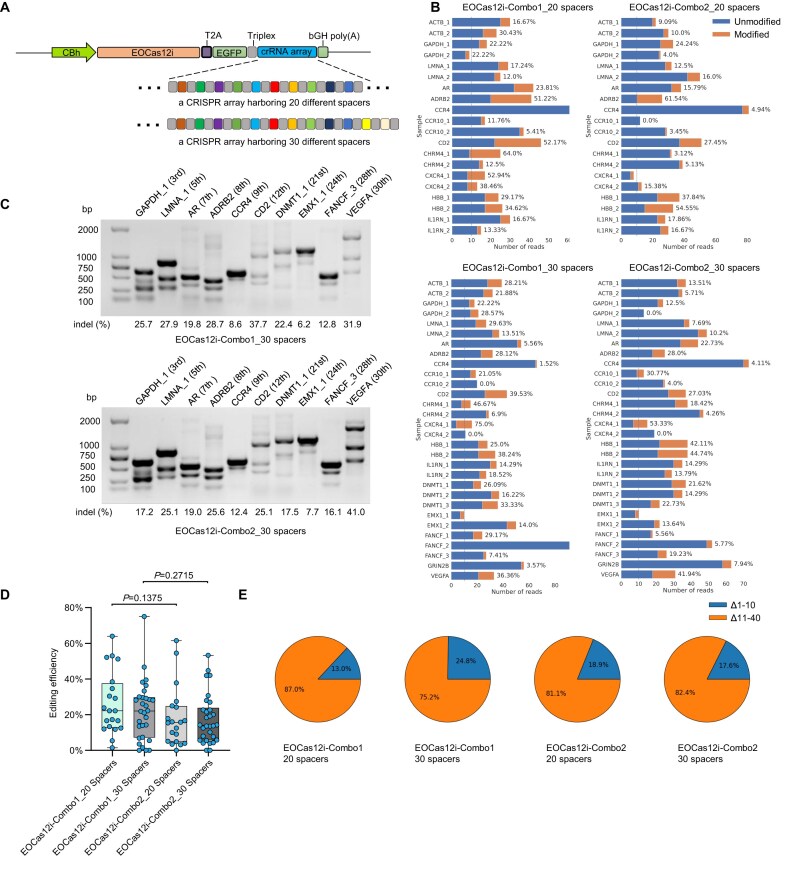
Multiplexed genome editing with EOCas12i. (**A**) Schematic of a single transcript encoding either EOCas12i–Combo1 or EOCas12i–Combo2 along with a CRISPR array. Two arrays are shown here, one CRISPR array containing 20 spacers and the other 30 spacers. In the CRISPR array, direct repeats are represented by gray squares, while spacers are indicated by colored squares. (**B**) Editing activity of EOCas12i–Combo1 and EOCas12i–Combo2 at 20 or 30 targets within a CRISPR array in HEK293T cells. Results were obtained through whole-genome sequencing (100 × coverage), followed by alignment, sorting, deduplication, and CRISPRessoWGS analysis. Detailed methods are described in the Materials and Methods section. Target positions are listed from first to last within the CRISPR array. (**C**) T7E1 cleavage gel images for EOCas12i–Combo1 and EOCas12i–Combo2 at positions 3, 5, 7, 8, 9, 12, 21, 24, 28, and 30 on the 30-spacer array in HEK293T cells. (**D**) Comparison of indel activities of EOCas12i–Combo1 and EOCas12i–Combo2 across multiple target sites arranged in tandem within 20- or 30-spacer CRISPR arrays in HEK293T cells. Significant difference was determined by unpaired Student’s *t*-test. (**E**) Genomic deletions induced by EOCas12i–Combo1 and EOCas12i–Combo2 across multiple target sites arranged in tandem within 20- and 30-spacer CRISPR arrays in HEK293T cells.

We evaluated the multiplexed genome editing capabilities of the EOCas12i platform using two CRISPR arrays: a 20-target array (12 loci: ACTB, GAPDH, LMNA, AR, ADRB2, CCR4, CCR10, CD2, CHRM4, CXCR4, HBB, and IL1RN) and a 30-target array (17 loci: ACTB, GAPDH, LMNA, AR, ADRB2, CCR4, CCR10, CD2, CHRM4, CXCR4, HBB, IL1RN, DNMT1, EMX1, FANCF, GRIN2B, and VEGFA). These arrays were positioned downstream of the EOCas12i coding sequence in a triplex structure (Fig. 7A and Supplementary Table S6). WGS followed by CRISPRessoWGS analysis was used to assess editing efficiency of EOCas12i–Combo1 and EOCas12i–Combo2 in HEK293T cells for both arrays. The results revealed consistently high editing efficiency at all target loci, regardless of whether a 20-target or 30-target array was used, or whether the vector was EOCas12i–Combo1 or EOCas12i–Combo2 (Fig. 7B). Specifically, T7E1 assays were conducted at positions 3, 5, 7, 8, 9, and 12 for the 20-target array, and at positions 3, 5, 7, 8, 9, 12, 21, 24, 28, and 30 for the 30-target array. Efficient cleavage was observed at all loci, except at position 5 of the 20-target array in EOCas12i–Combo1. Notably, the crRNA at position 30 of the 30-target array achieved editing efficiencies of 31.9% and 41.0% for EOCas12i–Combo1 and EOCas12i–Combo2, respectively (Fig. 7C and Supplementary Fig. S13). Statistical analysis revealed that EOCas12i–Combo1 generally exhibited higher average editing efficiency compared to EOCas12i–Combo2 for both the 20- and 30-target arrays (Fig. 7D). When the first 20 targets of the 30-target array were compared to the 20-target array, no significant difference in editing efficiency was observed, suggesting that the number of targets in the latter positions of the array does not substantially affect editing efficiency at the earlier loci (data not shown). Despite the multiplexed genome editing involving up to 30 targets, both vectors primarily generated long indels (>10 bp), similar to those observed in single-gene editing (Fig. 7E). These results confirm that both EOCas12i–Combo1 and EOCas12i–Combo2 are effective for multiplexed genome editing.

## Discussion

Many Cas proteins exhibit editing activity; however, only SpCas9 and AsCas12a are widely utilized, with editing efficiency often being a major limiting factor. To improve the editing efficiency of CRISPR–Cas12i.3, we first optimized crRNA in three key aspects and introduced two non-amino acid modifications to the Cas12i.3 nuclease. These optimized components were integrated to form the IOCas12i system, which demonstrated a significant increase in editing efficiency (2.0- to 3.9-fold) in sheep fibroblasts, NIH-3T3 cells, and HEK293T cells.

Amino acid mutations represent another crucial strategy to enhance CRISPR–Cas system efficiency. In this study, mutations were introduced to Cas12i.3, and combinations were made across different regions. These mutations were incorporated into the IOCas12i system to develop the EOCas12i system, which comprises two variants: EOCas12i–Combo1 and EOCas12i–Combo2. Both variants significantly improved editing efficiencies, ranging from 2.5- to 22.8-fold and 3.0- to 60.0-fold those of Cas12i.3 for Combo1 and Combo2, respectively. Compared to IOCas12i, their editing efficiencies ranged from 1.2- to 3.1-fold and 1.1- to 8.1-fold for Combo1 and Combo2, respectively. Notably, the editing efficiency of EOCas12i–Combo1 and EOCas12i–Combo2 at tested targets was comparable to that of SpCas9 and LbCas12a.

Several CRISPR–Cas effectors, including SpCas9, AaCas12b, AsCas12f, and LbCas12g, exhibit nuclease activity but lack the ability to autonomously process pre-crRNA and require tracrRNA for function [1]. Here, we achieved simultaneous editing of up to 30 targets by directly linking compact crRNAs targeting multiple genes, simplifying gRNA design and minimizing complexity. This achievement is likely attributable to engineering advancements that greatly enhanced the editing efficiency of Cas12i.3, as prior research in plants suggested that Cas12i.3 was incapable of processing pre-crRNA [13]. This approach facilitates coordinated gene deletions, activations, and repressions by adjusting the number and type of targets in the CRISPR array. This efficient multiplex gene-editing capability holds promise for advancing large-scale functional screening, gene regulation, and genomics studies in basic research, facilitating treatments for polygenic diseases in medicine, and supporting crop improvement by enabling the simultaneous enhancement of traits such as disease resistance, yield, and drought tolerance in agriculture. Previously, multiplexing strategies relied on heterologous expression of Csy4 for gRNA array processing, which often caused severe cytotoxicity, or on upstream promoters such as U6 to drive multiplex arrays, and on RNA elements like tRNAs and ribozymes to process crRNA array, both of which introduced significant complexity and, as a result, limited the practical applicability of both approaches [4, 13, 27].

Off-target effects are a major challenge in the research and application of gene-editing tools, directly impacting their precision, safety, and utility. We assessed the off-target effects of the EOCas12i system using GUIDE-seq, finding significantly fewer off-target edits compared to SpCas9. This enhanced specificity likely arises from several distinct mechanistic features. First, EOCas12i utilizes a 23-nt guide RNA—longer than the canonical 20-nt used in SpCas9—thereby increasing base-pairing stringency and reducing tolerance for mismatches, especially in the seed region. Structural studies on Cas12i1 have demonstrated that the protein can accommodate crRNA–DNA heteroduplexes of up to 28 bp [28], suggesting further potential for specificity tuning through guide length extension. In addition, Cas12i’s activation may require more stable crRNA–DNA hybrid formation and stricter conformational transitions, collectively reducing the likelihood of promiscuous cleavage. While GUIDE-seq offers sensitive, genome-wide detection of off-targets, it may underrepresent editing events in heterochromatic or transcriptionally silent regions. Complementary approaches such as CIRCLE-seq or DISCOVER-seq could help provide a more comprehensive off-target landscape in future studies.

In conclusion, the optimized EOCas12i–Combo1 and EOCas12i–Combo2 systems exhibit high editing efficiency, specificity, and robust potential for multiplexed genome editing, positioning them as promising tools for applications ranging from functional genomics to therapeutic and agricultural innovations.

## Supplementary Material

gkaf806_Supplemental_File

## Data Availability

The raw sequencing data from targeted deep sequencing, whole-genome sequencing (WGS), and GUIDE-seq generated in our study have been deposited in the NCBI Sequence Read Archive (SRA) under the BioProject PRJNA1247221.
