# Supplementary material for: An engineered CRISPR–Cas12i tool for efficient multiplexed genome editing
Source: Nucleic Acids Res. 2025 Aug 27;53(16):gkaf806. doi: 10.1093/nar/gkaf806 (PMC12390750; doi:10.1093/nar/gkaf806)
Supplement: gkaf806_Supplemental_File [file gkaf806_supplemental_file.pdf]

Supplementary Figures

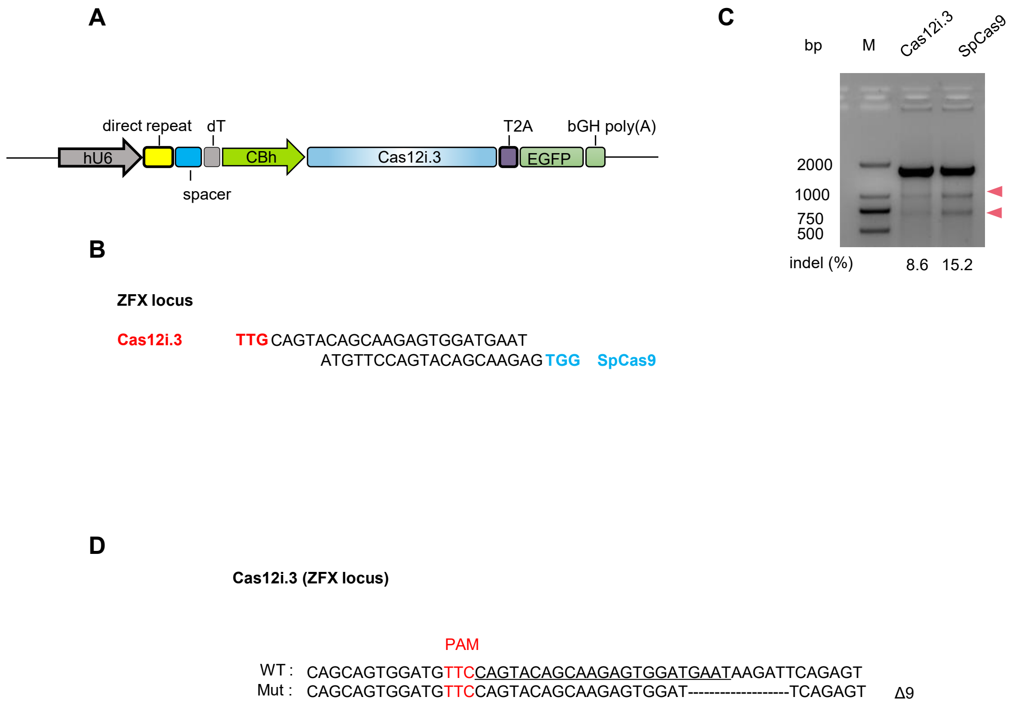

Figure S1. Cas12i.3, a type V Cas nuclease, exhibits low editing efficiency in animal cells. (A) Plasmid construct encoding Cas12i.3, EGFP, and crRNA. Cas12i.3 and EGFP are co-expressed from the CBh promoter via a T2A linker, while the crRNA is driven by the U6 promoter. (B) Sequence of the sheep ZFX gene protospacer that is targeted by Cas12i.3 and SpCas9. (C and D) Indel efficiency by Cas12i.3 at ZFX loci in sheep fibroblasts, determined by T7E1 assay (C), and TA cloning and sequencing (D). Red triangles indicate the cleaved bands. PAM sequences are marked in red. PAM, protospacer-adjacent motif. WT, wild-type. Mut, mutant.

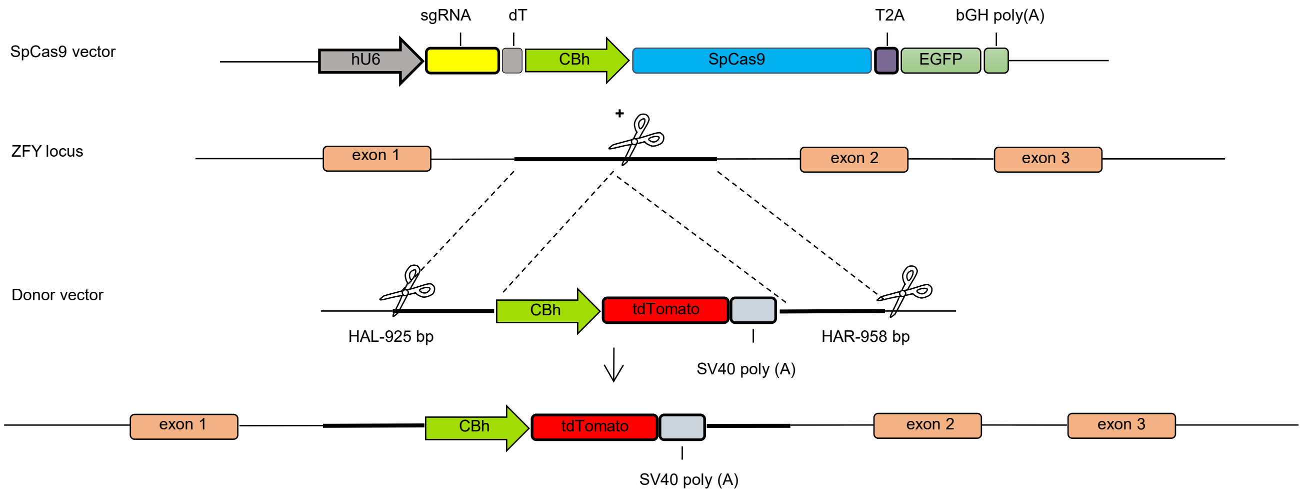

Figure S2. Experimental scheme for targeted CBh-tdTomato-SV40 poly(A) knock-in at the ZFY locus in sheep fibroblasts.

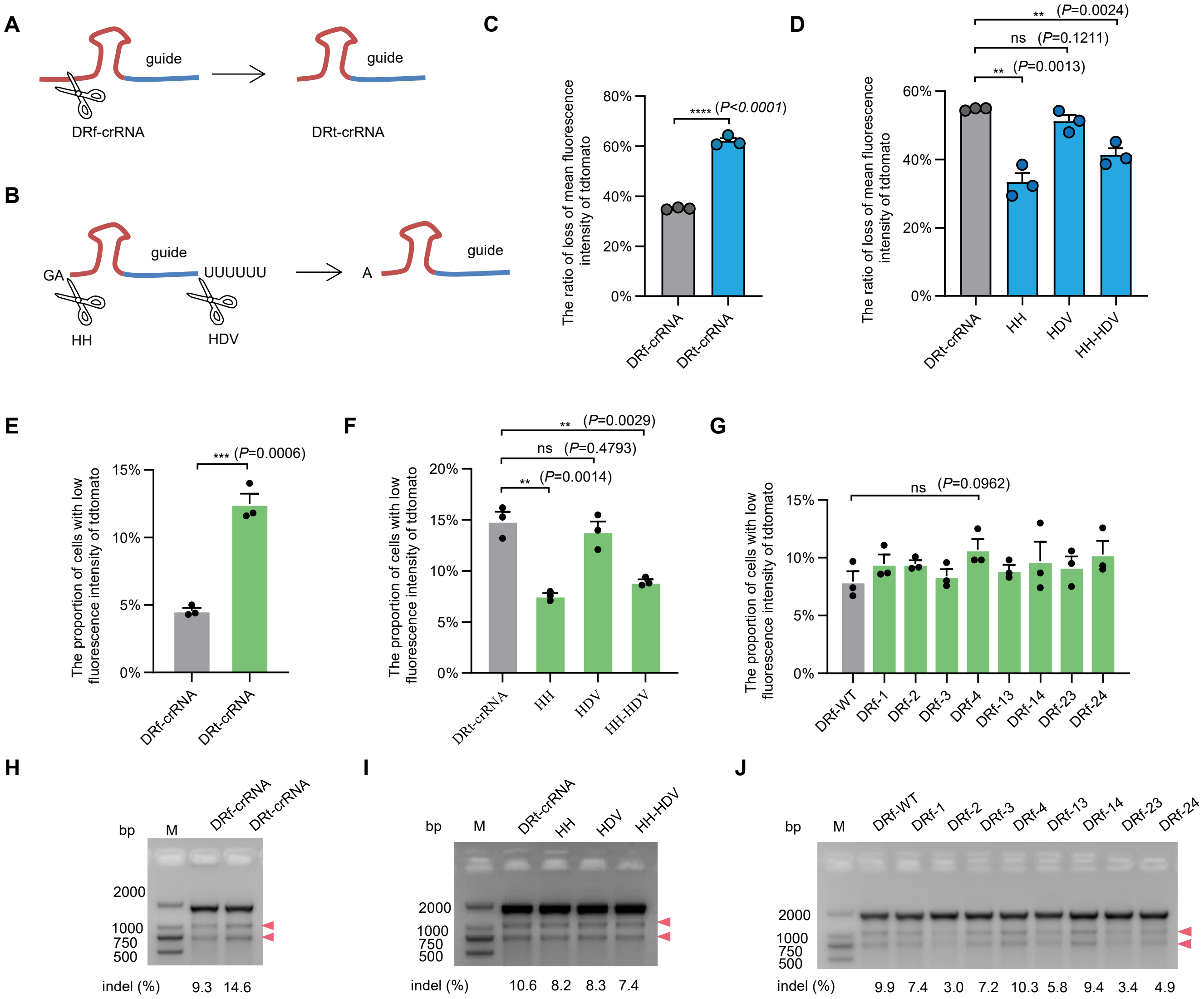

Figure S3. Optimization of Cas12i crRNA in sheep fibroblasts using three different approaches. (A) Schematic of full-length Direct-Repeat crRNA (DRf-crRNA) and truncated Direct-Repeat crRNA (DRt-crRNA). Repeat (red) and guide (blue) sequences are indicated. Scissors represent the Cas12i.3 RNase domain, which processes the crRNA at the repeat recognition sequence (RRS). (B) Schematic of precise expression of crRNA by hammerhead (HH) and/or hepatitis delta virus (HDV) ribozymes. Transcription of crRNA from the Pol III promoter is accompanied by unfavorable factors, such as a pyrimidine start nucleotide leading to inefficient expression and variable U1-6 tail that affects the activity of the CRISPR/Cas system after transcription. Scissors represent either HH or HDV ribozymes, which process crRNA at the beginning and end of DRt-crRNA, respectively. (C,D) Disruption of tdTomato using Cas12i.3 and crRNAs engineered with different direct repeats (DRf-crRNA or DRt-crRNA) (C), or processed by HH and/or HDV ribozymes (D) in sheep fibroblasts (n = 3 samples). DRf-WT, wild-type DRf-crRNA. (E-G) The proportion of cells with low fluorescence intensity of tdTomato in sheep fibroblasts, determined by flow cytometry (n = 3 samples). Shown are results using DRf-crRNA and DRt-crRNA (E), crRNAs processed by HH and/or HDV ribozymes (F), and stem-loop-engineered crRNAs (G). (H–J) Effect of crRNA design on Cas12i.3-mediated cleavage at the ZFX locus in sheep fibroblasts, determined by T7E1 assay. Shown are DRf-crRNA and DRt-crRNA (H), crRNAs processed by HH and/or HDV ribozymes (I), and stem-loop-engineered crRNAs (J). Red triangles indicate the cleaved bands. Mean±SE, ****P < 0.0001; ***P < 0.001; **P < 0.01; ns, not significant (unpaired Student's t-test).

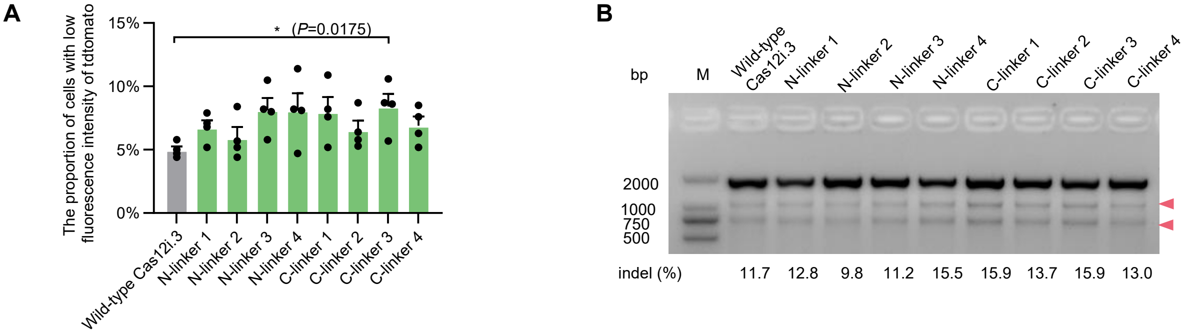

C

	GC%	CAI(human)	CAI(sheep)
Unoptimized	56.86%	0.83	0.75
Codon OptimWiz	58.01%	0.98	0.96
GeneOptimizer	56.19%	0.94	0.89
Jcat	63.22%	0.99	1.00
General Biol	53.67%	0.94	0.87

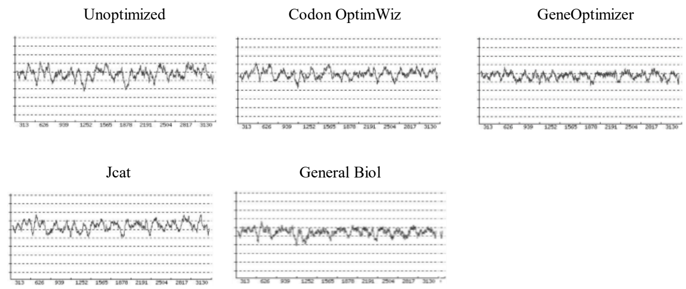

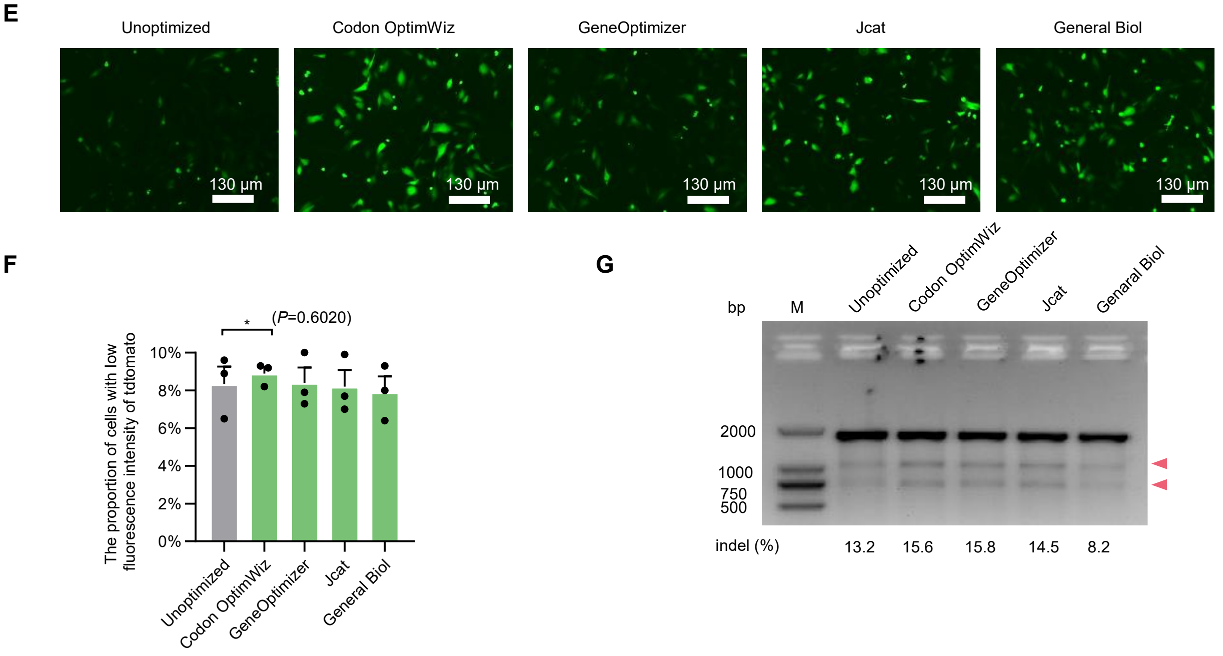

Figure S4. Effects of Cas12i.3 codon optimization and fusion of Cas12i.3 with exonuclease on CRISPR/Cas12i.3 cleavage activity in sheep fibroblasts. (A) The proportion of cells with low fluorescence intensity of tdTomato of Cas12i.3 fused with T5 exonuclease in different linkers in sheep fibroblasts, determined by flow cytometry (n = 4 samples). Mean±SE. (B) Effect of Cas12i.3 fused with T5 exonuclease in different linkers on cleavage activity at ZFX locus in sheep fibroblasts, determined by T7E1 assay. Red triangles indicate the cleaved bands. (C) The GC content of five sets of codons encoding Cas12i.3 and their codon adaptation index (CAI) in humans and sheep. (D) A distribution plot of the GC content for the five Cas12i.3 codon sequences. (E) Fluorescence images of sheep fibroblasts 2 days post-transfection with five codon-optimized Cas12i.3 in sheep fibroblast. Scale bar: 130 μm. (F) The proportion of cells with low fluorescence intensity of tdTomato of five different codon usages of Cas12i.3 in sheep fibroblasts, determined by flow cytometry (n = 3 samples). (G) Effect of five different codon usages of Cas12i.3 on cleavage activity at ZFX locus in sheep fibroblasts, determined by T7E1 assay. Mean±SE, **P* < 0.05 (unpaired Student's *t*-test).

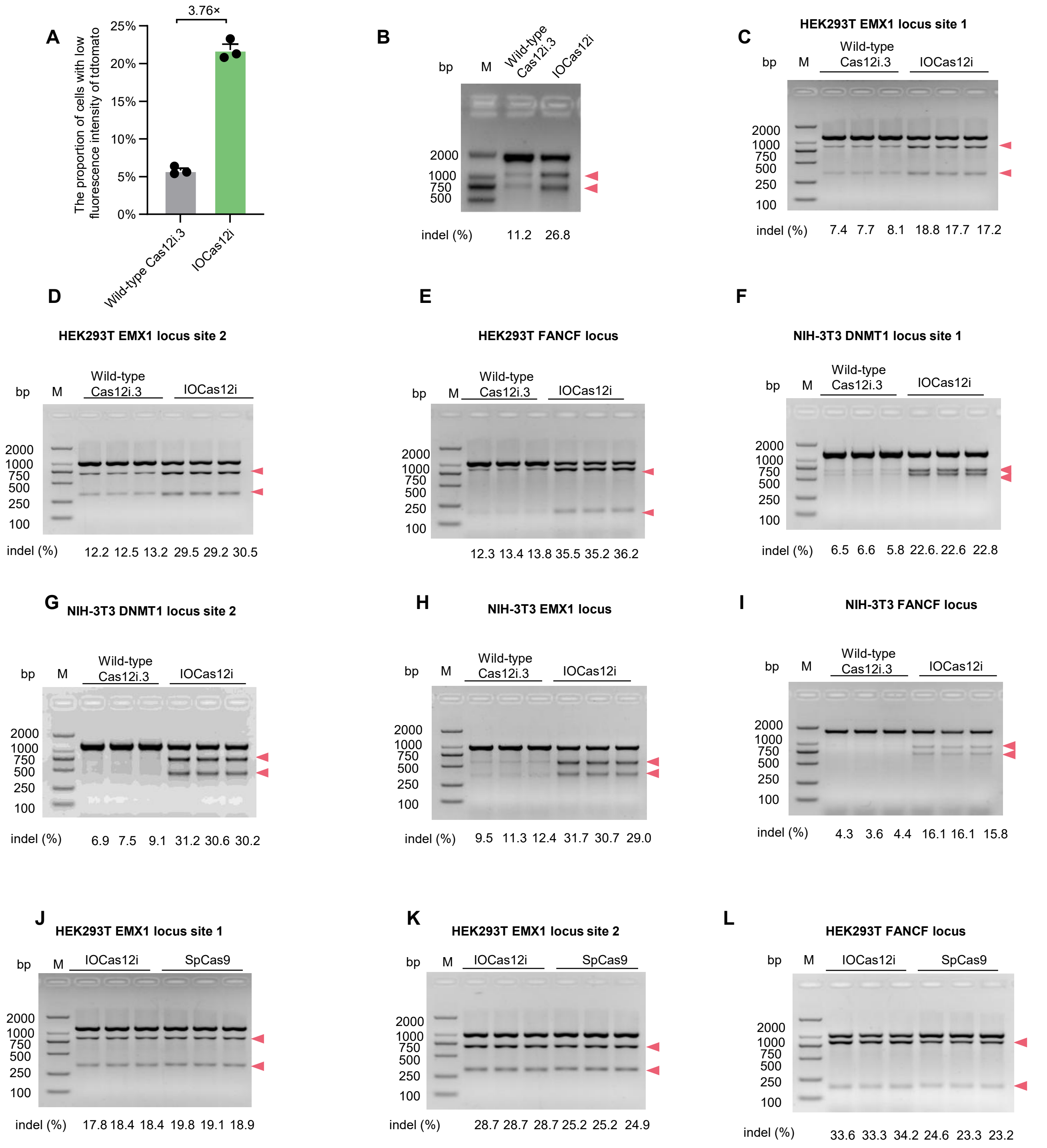

Figure S5. Enhancement of CRISPR/Cas12i.3 genome editing efficiency through the integration of two optimization steps. (A) The proportion of cells with low fluorescence intensity of tdTomato of IOCas12i in sheep fibroblasts, determined by flow cytometry (n = 3 samples). (B) Effect of IOCas12i on cleavage activity at ZFX locus in sheep fibroblasts, determined by T7E1 assay. Red triangles indicate the cleaved bands. (C-E) Indel frequencies of wild-type Cas12i.3 and IOCas12i at EMX1 and FANCF loci in HEK293T cells, determined by T7E1 assay (n = 3 samples). (F-I) Indel frequencies of wild-type Cas12i.3 and IOCas12i at DNMT1, EMX1, and FANCF loci in NIH-3T3 cells, determined by T7E1 assay (n = 3 samples). (J-L) Indel frequencies of IOCas12i and SpCas9 at EMX1 and FANCF loci in HEK293T cells, determined by T7E1 assay (n = 3 samples).

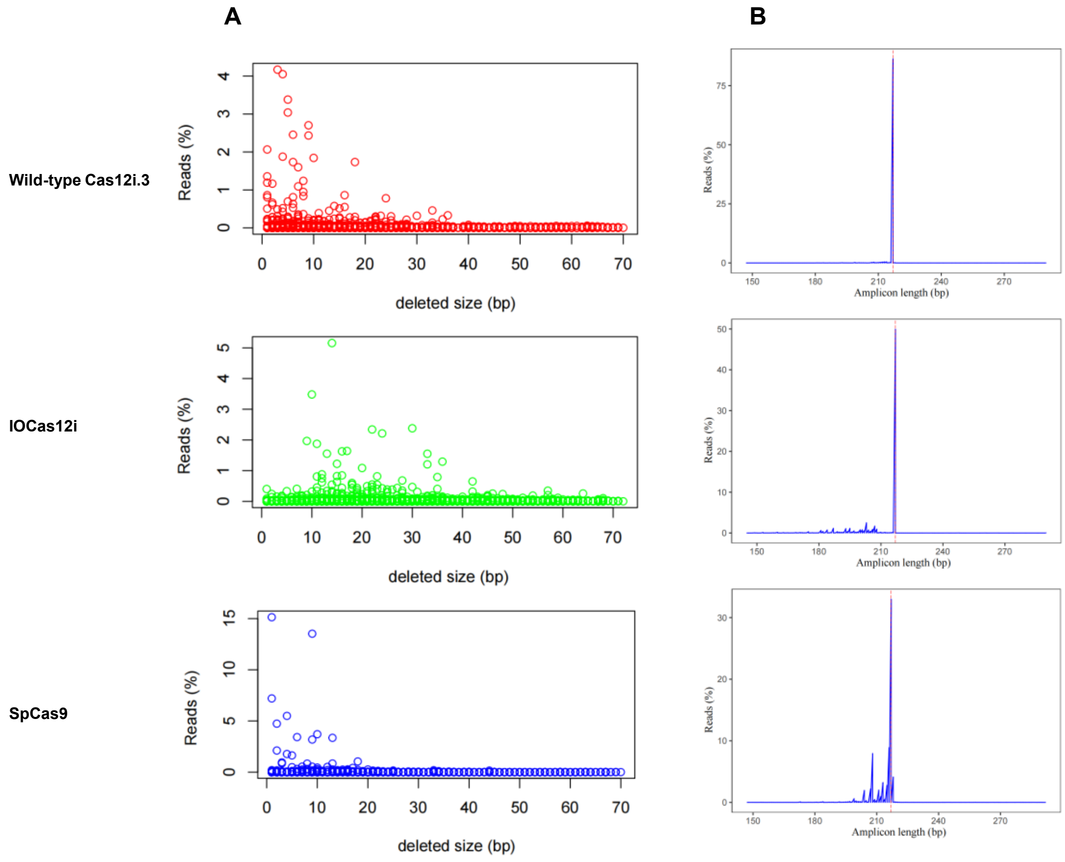

C

Wild-type Cas12i.3	Read(%)
TTACCATAGAGTCCTTGGTGGCCAAGGACGGCGGCACCGGCGG	86.33
TTACCATAGAGTCCTTGG---CCAAGGACGGCGGCACCGGCGG	0.49
TTACCATAGAGTCCTTG----CCAAGGACGGCGGCACCGGCGG	0.48
TTACCATAGAGTCCTTG-----CAAGGACGGCGGCACCGGCGG	0.40
TTACCATAGAGTCCTT-----CCAAGGACGGCGGCACCGGCGG	0.36
TTACCATAGAGTCCTTGG---------ACGGCGGCACCGGCGG	0.32
TTACCATAGAGTCCTT------CAAGGACGGCGGCACCGGCGG	0.29
TTACCATAGAGTCC---------AAGGACGGCGGCACCGGCGG	0.29
TTACCATAGAGTCCTTGGTGGCCAAG-ACGGCGGCACCGGCGG	0.24
TTACCATAGAGTCCTTGG----CAAGGACGGCGGCACCGGCGG	0.22
TTACCATAGAGTCCTTG----------ACGGCGGCACCGGCGG	0.22

IOCas12i	Read(%)
TTACCATAGAGTCCTTGGTGGCCAAGGACGGCGGCACCGGCGG	49.94
TTACCATAGAGTCCTTG--------------CGGCACCGGCGG	2.53
TTACCATAGAGTCCTTG----------ACGGCGGCACCGGCGG	1.71
TTACCA------------------------------CCGGCGG	1.17
TTACCATAG----------------------CGGCACCGGCGG	1.15
TTACCATAGAGTCC------------------------GGCGG	1.09
TTACCATAGAGTCCTTGG---------ACGGCGGCACCGGCGG	0.97
TTACCATAGAGTCCT-----------GACGGCGGCACCGGCGG	0.92
TTACCATAGAGTCCTTG-----------------CACCGGCGG	0.81
TTACCATAGAGTCCTTGG----------------CACCGGCGG	0.80
TTACCATAGAGTCCTTGG-------------CGGCACCGGCGG	0.76

SpCas9	Read(%)
TTACCATAGAGTCCTTGGTGGCCAAGGACGGCGGCACCGGCGG	32.99
TTACCATAGAGTCCTTGGTG-CCAAGGACGGCGGCACCGGCGG	8.90
TTACCATAGAGTCC---------AAGGACGGCGGCACCGGCGG	7.95
TTACCATAGAGTCCTTGGTGGC-AAGGACGGCGGCACCGGCGG	4.24
TTACCATAGAGTCCTTG----CCAAGGACGGCGGCACCGGCGG	3.23
TTACCATAGAGTCCTTGGTGG–AAGGACGGCGGCACCGGCGG	2.78
TTACCATAGAGTCCTTGGTGGC----------GGCACCGGCGG	2.18
TTACCATAGAGTCCTTGGTGG------ACGGCGGCACCGGCGG	2.01
TTACCATAGAGTCCTTGGTGGC-------------ACCGGCGG	1.97
TTACCATAGAGTCCTTGG---------ACGGCGGCACCGGCGG	1.87
TTACCATAGAGTCCTTGGT--CCAAGGACGGCGGCACCGGCGG	1.24

Figure S6. Indel size distribution and position distribution induced by Cas12i.3, IOCas12i and SpCas9 system. (A) Indel size distribution induced by wild-type Cas12i.3, IOCas12i, and SpCas9 system in HEK293T cells. (B) Position distribution induced by wild-type Cas12i.3, IOCas12i and SpCas9 system in HEK293T cells. The red dashed line indicates the predicted cleavage position. (C) Representative top ten high-frequent on-target mutagenesis aligned to the target site of EMX1 locus induced by wild-type Cas12i.3, IOCas12i, and SpCas9 system in HEK293T cells. The protospacer-adjacent motif is highlighted in red (wild-type Cas12i.3 and IOCas12i) or blue (SpCas9).

A

PAM

WT: TTC<u>AGAAGGGGCTTTGTAGATGACCT</u>TGACCTTGCTGT
KO #1: TTCAGAAGGGGCTTTGTAGA------------------CCTTGCTGT Δ9
KO #2: TTCAGAAGGGGCTTTG------------------------------------CTGT Δ18

B

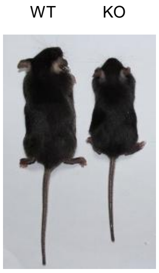

C

PAM

WT: TTC<u>AGAAGGGGCTTTGTAGATGACCT</u>TGACCTTGCTGT
KO: TTCAGAAGGGGCTTTG------------------------------------CTGT Δ18

Figure S7. IOCas12i enables genome-editing in mouse embryos and generation of gene-edited animals. (A) IOCas12i-mediated gene-editing at DNMT1 locus in mouse embryo, determined by TA cloning and sequencing. WT, wild-type mouse embryo. KO, DNMT1-knockout mouse embryo. (B) Phenotypic differences between IOCas12i-meditated DNMT1 gene-edited mouse and wild-type mouse at 7 weeks. WT, wild-type mouse. (C) IOCas12i-mediated editing at the DNMT1 locus in C57 mouse, determined by TA cloning and sequencing. PAM sequences are marked in red. PAM, protospacer-adjacent motif. WT, wild-type mouse. KO, DNMT1-knockout mouse.

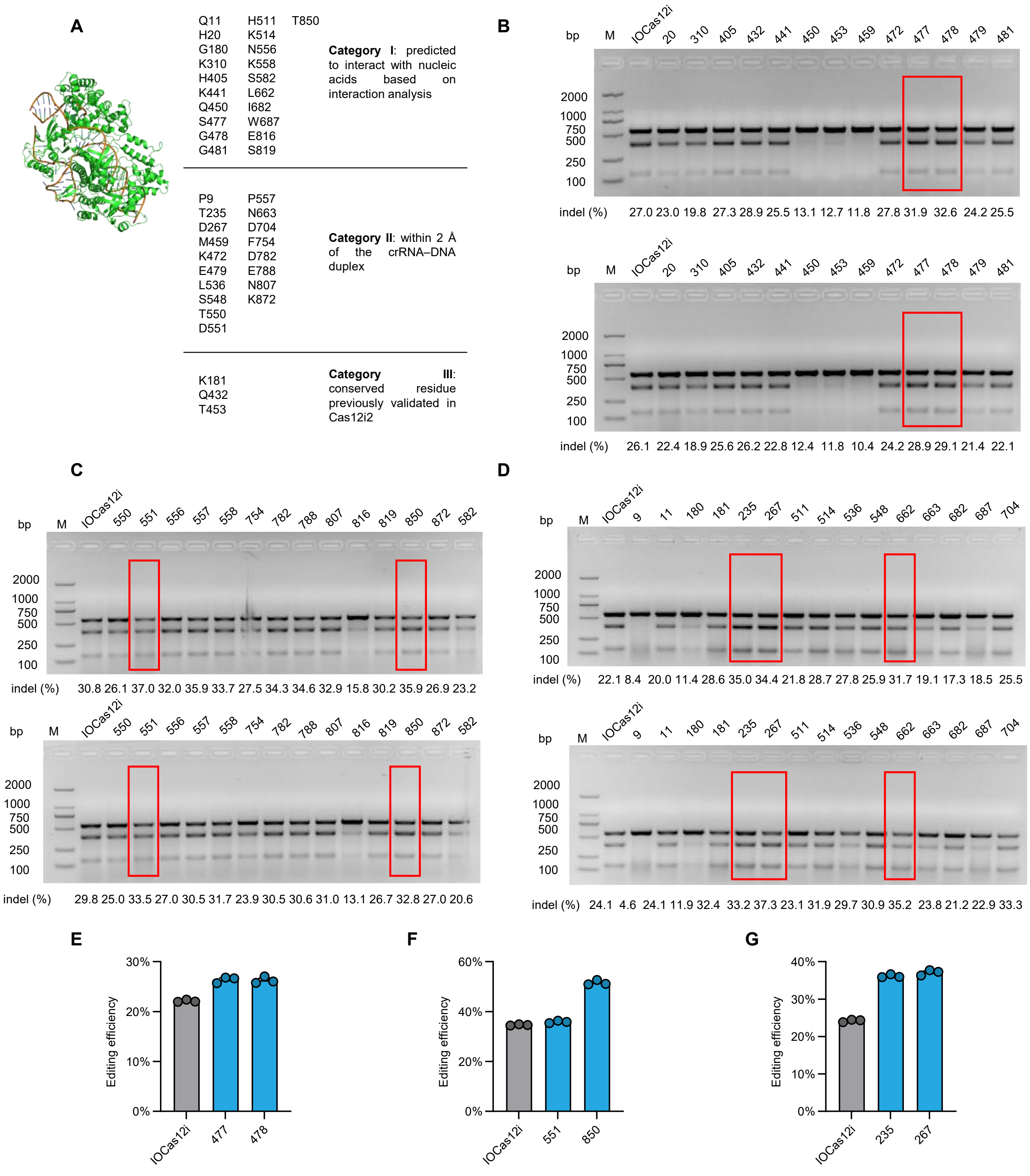

Figure S8. Construction of efficient single-mutation IOCas12i variants. (A) Potential mutation sites in Cas12i.3 were identified through the analysis of its 3D-reconstructed ternary complex with crRNA and DNA. (B-D) The editing efficiency of single-mutation IOCas12i variants in HEK293T cells, determined by T7E1 assay (n = 2 samples). Mutations conferring the highest increase in editing efficiency in each experimental group are highlighted in the red rectangular. Numbers (e.g., 20 and 310) represent the positions of amino acid residues in the protein sequence that are mutated to arginine (R). (E–G) Validation of editing efficiency for the two top-performing single-mutation IOCas12i variants from each group identified in B–D, assessed by targeted deep sequencing in HEK293T cells (n = 3 samples). The sequencing results are consistent with the T7E1 assay and confirm enhanced editing by selected variants.

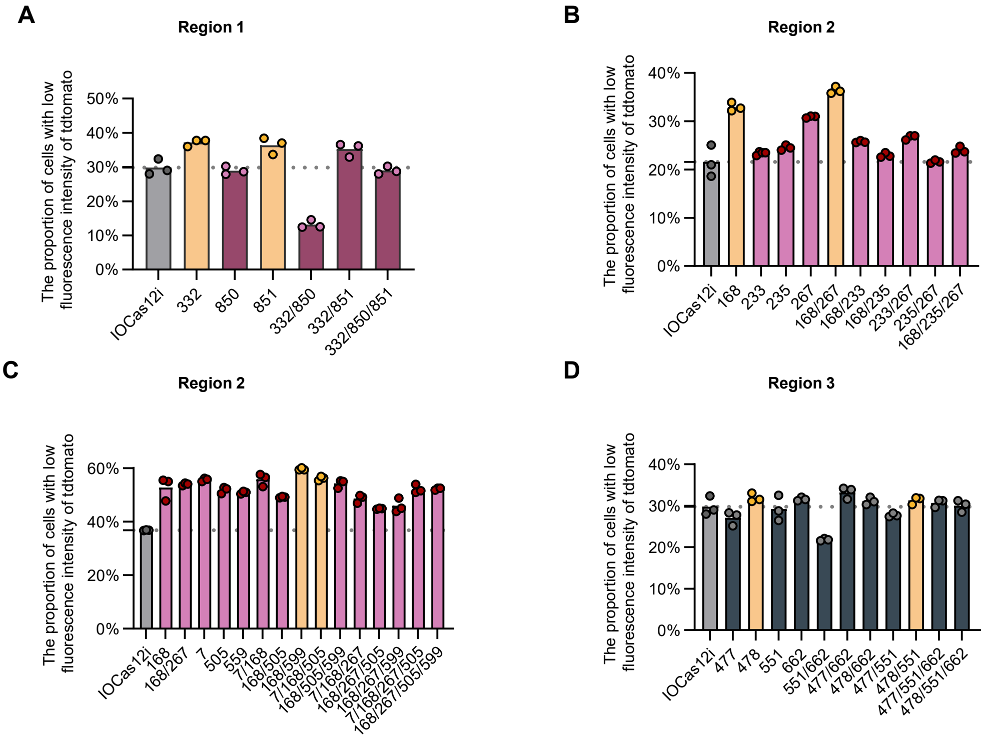

Figure S9. The proportion of cells with low tdTomato fluorescence intensity in different mutation combinations across IOCas12i regions, as assessed by flow cytometry in sheep fibroblasts. Panels (A), (B and C), and (D), correspond to regions 1, 2, and 3, respectively. The final combination is based on two mutation combinations from S7R/N168R/T505R and N168R/S599R from Region 2, which showed the greatest enhancement in efficiency. Numbers (e.g., 332 and 850) represent the positions of amino acid residues in the protein sequence that are mutated to arginine (R). n = 3 samples.

Build the final variant based on 168/599

	Region 2	Region 1	Region 3	Final variant
	168/599			168/599
1-1	168/599	332		168/332/599
1-2	168/599	851		168/599/851
1-3	168/599		478	168/478/599
1-4	168/599		478/551	168/478/551/599
1-5	168/599	332	478	168/332/478/599
1-6	168/599	332	478/551	168/332/478/551/599
1-7	168/599	851	478	168/478/599/851
1-8	168/599	851	478/551	168/478/551/599/851

Build the final variant based on 7/168/505

	Region 2	Region 1	Region 3	Final variant
	7/168/505			7/168/505
2-1	7/168/505	332		7/168/332/505
2-2	7/168/505	851		7/168/505/851
2-3	7/168/505		478	7/168/478/505
2-4	7/168/505		478/551	7/168/478/505/551
2-5	7/168/505	332	478	7/168/332/478/505
2-6	7/168/505	332	478/551	7/168/332/478/505/551
2-7	7/168/505	851	478	7/168/478/505/851
2-8	7/168/505	851	478/551	7/168/478/505/551/851

Figure S10. Scheme for further combining of mutation combinations identified from the three-region screen to generate variants with higher editing efficiency. Numbers (e.g., 168/599 and 332) represent the positions of amino acid residues in the protein sequence that are mutated to arginine (R).

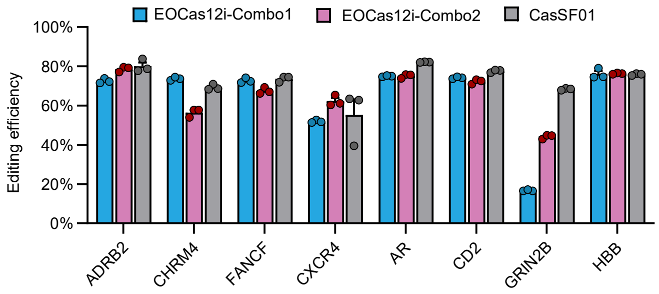

Figure S11. Indel efficiencies by EOCas12i-Combo1, EOCas12i-Combo2, CasSF01 at eight endogenous target loci in HEK293T cells (n = 3 samples), determined by targeted deep sequencing.

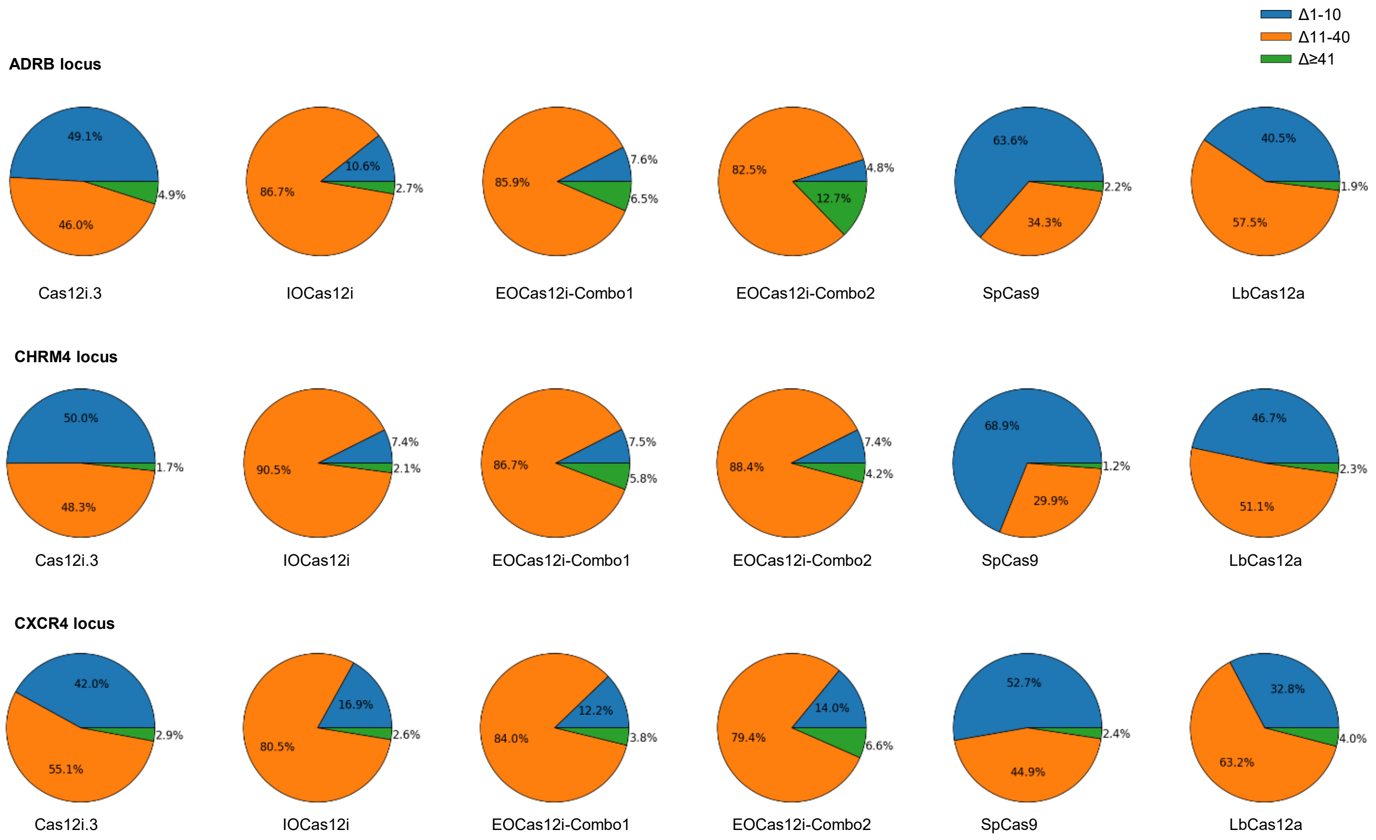

Figure S12. EOCas12i induces longer genomic deletions at the ADRB2, CHRM4, and CXCR4 loci than Cas12i.3, SpCas9, and LbCas12a in HEK293T cells.

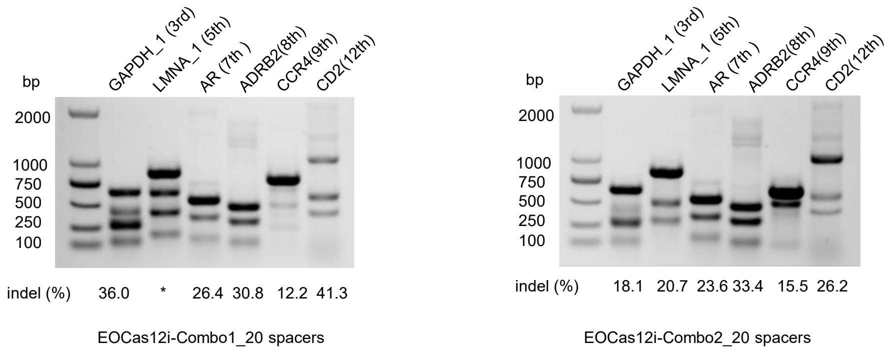

Figure S13. T7E1 cleavage gel images for EOCas12i-Combo1 and EOCas12i-Combo2 at positions 3, 5, 7, 8, 9, and 12 on the 20-spacer array in HEK293T cells.

Supplementary Tables

Table S1

**Sequence**	
Cas12i.3 protein	MKKVEVSRPYQSLLLPNHRKFKYLDETWNAYKSVKSLLHRFLVCAYGAVPFN<br>KFVEVVEKVDNDQLVLAFAVRLFRLVPVESTSFAKVDKANLAKSLANHLPVGT<br>AIPANVQSYFDSNFDPKKYMWIDCAWEADRLAREMGLSASQFSEYATTMLW<br>EDWLPLNKDDVNGWGSVSGLFGEGKKEDRQQKVKMLNNLLNGIKKNPPKD<br>YTQYLKILLNAFDAKSHKEAVKNYKGDSTGRTASYLSEKSGEITELMLEQLMS<br>NIQRDIGDKQKEISLPKKDVVKKYLESESGVPYDQNLWSQAYRNAASSIKKTD<br>TRNFNSTLEKFKNEVELRGLLSEGDDVEILRSKFFSSEFHKTPDKFVIKPEHIG<br>FNNKYNVVAELYKLKAEATDFESAFATVKDEFEEKGIKHPIKNILEYIWNNEVP<br>VEKWGRVARFNQSEEKLLRIKANPTVECNQGMTFGNSAMVGEVLRSNYVS<br>KKGALVSGEHGGRLIGQNNMIWLEMRLLNKGKWETHHVPTHNMKFFEEVH<br>AYNPSLADSVNVRNRLYRSEDYTQLPSSITDGLKGNPKAKLLKRQHCALNNM<br>TANVLNPKLSFTINKKNDDYTVIIVHSVEVSKPRREVLVGDYLVGMDQNQTAS<br>NTYAVMQVVKPKSTDAIPFRNMWVRFVESGSIESRTLNSRGEYVDQLNHDG<br>VDLFEIGDTEWVDSARKFFNKLGVKHKDGTLVDLSTAPRKAYAFNNFYFKTM<br>LNHLRSNEVDLTLLRNEILRVANGRFSPMRLGSLSWTTLKALGSFKSLVLSYF<br>DRLGAKEMVDKEAKDKSLFDLLVAINNKRSNKREERTSRIASSLMTVAQKYK<br>VDNAVVHVVVEGNLSSTDRSASKAHNRNTMDWCSRAVVKKLEDMCNLYGF<br>NIKGVPAFYTSHQDPLVHRADYDDPKPALRCRYSSYSRADFSKWGQNALAA<br>VVRWASNKKSNTCYKVGAVEFLKQHGLFADKKLTVEQFLSKVKDEEILIPRR<br>GGRVFLTTHRLLAESTFVYLNGVKYHSCNADEVAAVNICLNDWVIPCKKKMK<br>EESSASG
Cas12i.3_coding sequence_Unoptimized	ATGAAGAAGGTCGAGGTGTCGCGGCCATACCAGAGCCTGCTCCTGCCAA<br>ACCACCGGAAGTTCAAGTACCTCGACGAGACCTGGAATGCGTACAAGTC<br>CGTTAAGAGCCTGCTCCACCGCTTCCTGGTGTGCGCTTACGGCGCTGTT<br>CCCTTCAACAAGTTCGTGGAGGTTGTCGAGAAGGTTGATAACGACCAGCT<br>CGTGCTGGCTTTCGCGGTGCGCCTCTTCCGCCTGGTCCCCGTGGAGAG<br>CACCTCTTTCGCCAAGGTTGACAAGGCCAATCTGGCGAAGTCCCTGGCC<br>AATCACCTGCCTGTGGGCACAGCCATTCCTGCCAATGTTCAGTCCTACTT<br>CGATTCAAATTTCGACCCCAAGAAGTACATGTGGATCGACTGCGCGTGGG<br>AGGCTGATCGCCTGGCTCGGGAGATGGGCCTGAGCGCGAGCCAGTTCT<br>CTGAGTACGCGACTACAATGCTCTGGGAGGACTGGCTGCCCCTCAATAAG<br>GATGATGTGAACGGCTGGGGGTCCGTGTCGGGGCTCTTCGGCGAGGGC<br>AAGAAGGAGGACCGGCAGCAGAAGGTGAAGATGCTGAATAACCTGCTGA<br>ATGGCATCAAGAAGAATCCGCCCAAGGATTACACCCAGTACCTGAAGATC<br>CTCCTGAACGCGTTCGACGCGAAGTCGCACAAGGAGGCTGTTAAGAACT<br>ACAAGGGGGACTCTACGGGGCGCACCGCGTCTTACCTGTCAGAGAAGTC<br>TGGCGAGATCACAGAGCTGATGCTCGAGCAGCTGATGTCAAACATCCAG<br>AGGGATATTGGCGACAAGCAGAAGGAGATCTCCCTGCCGAAGAAGGACG<br>TGGTCAAGAAGTACCTCGAGTCAGAGTCCGGCGTCCCATACGATCAGAA

	CCTGTGGTCCCAGGCCTACCGCAACGCTGCCAGCTCGATCAAGAAGACT<br>GATACGCGGAACTTCAACTCCACTCTCGAGAAGTTCAAGAATGAGGTGGA<br>GCTGAGGGGCCTGCTGAGCGAGGGCGACGACGTTGAGATCCTGAGGTC<br>TAAGTTCTTCAGCAGCGAGTTCCACAAGACCCCTGATAAGTTCGTTATTAA<br>GCCAGAGCATATTGGGTTCAACAATAAGTACAATGTCGTTGCCGAGCTGTA<br>CAAGCTCAAGGCTGAGGCGACCGATTTCGAGAGCGCTTTCGCCACAGTC<br>AAGGATGAGTTCGAGGAGAAGGGCATCAAGCACCCAATCAAGAACATCCT<br>CGAGTACATCTGGAATAACGAGGTGCCCGTCGAGAAGTGGGGCCGGGTT<br>GCCCGCTTCAACCAGTCCGAGGAGAAGCTCCTCCGGATTAAGGCCAACC<br>CCACGGTGGAGTGCAACCAGGGCATGACCTTCGGCAATTCCGCGATGGT<br>TGGCGAGGTCCTCAGGTCCAACTACGTCTCTAAGAAGGGCGCGCTGGTG<br>TCCGGCGAGCACGGCGGGCGCCTGATCGGCCAGAACAATATGATCTGGC<br>TGGAGATGCGGCTGCTCAACAAGGGGAAGTGGGAGACCCACCACGTTC<br>CAACCCATAACATGAAGTTCTTCGAGGAGGTGCATGCCTACAATCCCTCT<br>CTGGCGGATTCTGTTAACGTGCGGAATCGGCTGTACCGCTCAGAGGACTA<br>CACCCAGCTGCCTTCAAGCATTACCGACGGGCTGAAGGGCAATCCGAAG<br>GCGAAGCTCCTGAAGCGCCAGCACTGCGCTCTGAACAATATGACAGCTA<br>ATGTTCTCAATCCTAAGCTGAGCTTCACGATCAACAAGAAGAACGATGATT<br>ACACGGTCATCATTGTCCATAGCGTTGAGGTCTCGAAGCCTCGGAGGGA<br>GGTGCTCGTTGGCGATTACCTCGTGGGCATGGACCAGAATCAGACAGCG<br>TCTAATACATACGCCGTCATGCAGGTCGTCAAGCCGAAGTCTACAGATGC<br>GATCCCGTTCCGCAACATGTGGGTGCGGTTCGTGGAGTCAGGGTCTATC<br>GAGTCCCGGACCCTCAACAGCCGCGGGGAGTATGTTGATCAGCTGAATC<br>ATGACGGCGTGGACCTCTTCGAGATCGGCGATACGGAGTGGGTGGACTC<br>CGCGCGGAAGTTCTTCAATAAGCTCGGCGTTAAGCACAAGGATGGCACA<br>CTGGTTGATCTGTCTACGGCGCCCCGGAAGGCCTACGCTTTCAACAACTT<br>CTACTTCAAGACCATGCTGAATCATCTCCGGAGCAATGAGGTTGACCTGA<br>CGCTCCTGCGCAATGAGATCCTCCGGGTTGCCAATGGGCGGTTCTCCCC<br>GATGCGCCTCGGCTCGCTCTCCTGGACTACTCTCAAGGCCCTGGGCTCG<br>TTCAAGTCCCTGGTGCTGTCGTACTTCGACCGGCTGGGCGCCAAGGAGA<br>TGGTCGACAAGGAGGCTAAGGATAAGTCTCTCTTCGACCTCCTCGTGGCT<br>ATCAACAACAAGCGCTCTAATAAGCGCGAGGAGCGGACTTCCCGGATTGC<br>CTCCAGCCTCATGACTGTGGCGCAGAAGTACAAGGTTGATAACGCTGTG<br>GTCCATGTGGTCGTCGAGGGGAATCTCTCCAGCACGGACAGGAGCGCGT<br>CAAAGGCCCATAATCGGAACACTATGGATTGGTGCTCTAGGGCCGTGGTG<br>AAGAAGCTGGAGGACATGTGCAATCTCTACGGCTTCAATATCAAGGGCGT<br>CCCAGCCTTCTACACATCCCACCAGGACCCGCTCGTCCACCGCGCCGAC<br>TACGATGACCCTAAGCCGGCGCTCAGGTGCCGCTACTCCTCGTACTCAAG<br>GGCGGACTTCAGCAAGTGGGGGCAGAACGCTCTCGCGGCGGTGGTTCG<br>CTGGGCGTCTAATAAGAAGTCCAACACCTGCTACAAGGTCGGGGCCGTG<br>GAGTTCCTCAAGCAGCACGGCCTCTTCGCGGACAAGAAGCTGACAGTCG<br>AGCAGTTCCTCTCGAAGGTGAAGGACGAGGAGATCCTCATTCCCCGCAG<br>GGGCGGGAGGGTGTTCCTCACAACTCACCGGCTCCTGGCGGAGTCCAC<br>TTTCGTGTACCTGAACGGCGTTAAGTACCATTCATGCAACGCCGATGAGG

	TGGCGGCTGTTAACATCTGCCTGAATGACTGGGTTATCCCGTGCAAGAAG<br>AAGATGAAGGAGGAGTCAAGCGCGTCCGGG
Cas12i.3_<br>coding<br>sequence<br>_Codon<br>OptimWiz	ATGAAGAAGGTGGAGGTGAGCAGACCCTATCAGTCCCTGCTCCTGCCCA<br>ACCACAGAAAGTTCAAGTACCTGGACGAGACCTGGAACGCCTACAAGAG<br>CGTGAAAAGCCTGCTGCATAGATTCCTGGTGTGCGCCTACGGCGCCGTG<br>CCCTTCAACAAGTTCGTGGAGGTGGTGGAGAAAGTCGACAACGATCAAC<br>TGGTGCTGGCCTTCGCCGTGAGACTGTTCAGACTGGTGCCCGTGGAGA<br>GCACAAGCTTCGCCAAGGTGGACAAGGCCAACCTGGCCAAGAGCCTGG<br>CCAACCACCTGCCCGTGGGCACCGCCATCCCCGCTAACGTGCAGAGCTA<br>TTTCGACTCCAACTTCGACCCCAAGAAGTACATGTGGATCGACTGCGCCT<br>GGGAGGCCGACAGACTGGCTAGAGAGATGGGCCTGAGCGCTAGCCAATT<br>CAGCGAGTACGCCACCACCATGCTGTGGGAGGACTGGCTGCCCCTGAAC<br>AAGGACGACGTGAACGGCTGGGGCAGCGTGAGCGGCCTGTTCGGCGAG<br>GGCAAGAAGGAGGACAGACAGCAGAAGGTGAAGATGCTGAACAACCTG<br>CTGAACGGCATCAAGAAGAACCCTCCCAAGGACTACACACAGTACCTGAA<br>GATCCTGCTGAACGCCTTCGACGCCAAGAGCCACAAGGAGGCCGTGAA<br>GAACTACAAGGGCGACAGCACCGGCAGAACCGCTAGCTACCTGAGCGAG<br>AAGAGCGGCGAGATCACCGAGCTGATGCTGGAGCAGCTGATGAGCAACA<br>TTCAGAGAGACATCGGCGACAAGCAGAAGGAGATCAGCCTGCCCAAGAA<br>GGACGTGGTGAAAAAGTACCTGGAGAGCGAGAGCGGCGTGCCCTACGAT<br>CAGAACCTGTGGAGCCAAGCCTACAGAAACGCCGCTAGCTCCATTAAAAA<br>GACCGACACAAGAAACTTCAACAGCACCCTGGAGAAGTTCAAGAACGAG<br>GTGGAGCTGAGAGGCCTGCTGAGCGAGGGCGACGACGTGGAGATTCTG<br>AGATCCAAATTCTTCAGCAGCGAGTTCCACAAGACCCCCGACAAGTTCGT<br>GATCAAGCCCGAGCACATCGGCTTCAACAACAAGTACAACGTGGTGGCC<br>GAGCTGTACAAGCTGAAGGCCGAGGCCACCGACTTCGAGAGCGCCTTC<br>GCCACCGTGAAGGACGAGTTCGAGGAGAAGGGCATCAAGCACCCCATCA<br>AGAACATCCTGGAGTACATCTGGAACAACGAGGTGCCCGTGGAGAAATG<br>GGGCAGAGTGGCTAGATTCAATCAGAGCGAGGAGAAGCTGCTGAGAATC<br>AAGGCCAACCCCACCGTGGAGTGCAACCAAGGCATGACCTTCGGCAACA<br>GCGCCATGGTGGGCGAGGTGCTGCGGAGCAACTACGTGAGCAAGAAGG<br>GCGCCCTGGTGAGCGGCGAGCACGGCGGCAGACTGATCGGGCAGAACA<br>ACATGATCTGGCTGGAGATGAGACTGCTGAACAAGGGCAAGTGGGAGAC<br>CCACCACGTGCCCACCCACAACATGAAGTTCTTCGAAGAGGTGCACGCC<br>TACAACCCTAGCCTGGCCGACAGCGTGAACGTGAGAAACAGACTGTACA<br>GAAGCGAGGATTACACCCAACTCCCTAGCTCCATCACAGACGGCCTGAA<br>GGGCAACCCCAAGGCCAAGCTGCTGAAGAGACAGCACTGCGCCCTGAA<br>CAACATGACAGCCAACGTGCTGAACCCCAAGCTGAGCTTCACCATCAACA<br>AGAAGAACGACGACTACACCGTGATCATCGTGCACAGCGTCGAAGTGAG<br>CAAGCCTAGAAGAGAGGTGCTGGTGGGCGACTACCTGGTGGGCATGGAT<br>CAGAATCAGACCGCTAGCAACACCTACGCCGTGATGCAAGTGGTGAAGC<br>CCAAGAGCACCGACGCCATCCCCTTCAGAAACATGTGGGTGAGATTCGT<br>GGAGAGCGGCAGCATCGAGAGCAGAACCCTGAACAGCAGAGGCGAGTA<br>CGTGGATCAGCTGAACCACGACGGCGTGGACCTGTTCGAGATCGGCGAC

	ACCGAGTGGGTGGACAGCGCTAGAAAGTTCTTCAACAAGCTGGGCGTGAAGCACAAGGACGGCACCCTGGTGGACCTGAGCACCGCCCCTAGAAAGGCCTACGCCTTCAACAACTTCTACTTCAAGACCATGCTGAACCACCTCAGAAGCAACGAGGTCGACCTGACCCTGCTGAGAAACGAGATCCTGAGAGTGGCCAACGGCAGATTCAGCCCCATGAGACTGGGCAGCCTGAGCTGGACCACCCTGAAGGCCCTGGGCAGCTTCAAGAGCCTGGTGCTGAGCTACTTCGACAGACTGGGCGCCAAGGAGATGGTGGACAAGGAGGCCAAGGACAAGAGCCTGTTCGACCTGCTGGTGGCCATCAACAACAAGAGAAGCAACAAGAGAGAGGAGAGAACAAGCAGAATCGCTAGCAGCCTGATGACCGTGGCTCAGAAGTACAAAGTGGACAACGCCGTGGTGCACGTGGTCGTGGAGGGCAACCTGAGCAGCACCGACAGAAGCGCTAGCAAGGCCCACAACAGAAACACCATGGACTGGTGCAGCAGAGCCGTGGTGAAGAAGCTGGAGGACATGTGCAACCTGTACGGCTTCAACATCAAGGGCGTGCCCGCCTTCTACACAAGCCACCAAGACCCCCTGGTGCACAGAGCCGACTACGATGACCCCAAGCCCGCCCTGAGATGCAGATACAGCAGCTACAGCAGAGCCGACTTCAGCAAGTGGGGGCAGAACGCCCTGGCCGCCGTGGTGAGATGGGCTAGCAACAAGAAGAGCAACACCTGCTACAAGGTGGGCGCCGTGGAGTTCCTGAAGCAGCACGGCCTGTTCGCCGACAAGAAGCTGACCGTGGAGCAGTTCCTGAGCAAGGTGAAGGACGAAGAGATCCTGATCCCTAGAAGAGGCGGCAGAGTGTTCCTGACCACCCACAGACTGCTGGCCGAGAGCACCTTCGTGTACCTGAACGGCGTGAAGTACCACAGCTGCAACGCCGACGAGGTGGCCGCCGTGAACATCTGCCTGAACGACTGGGTGATCCCCTGCAAGAAAAAGATGAAGGAGGAGAGCAGCGCTAGCGGC
Cas12i.3_ coding sequence _GeneOp timizer	ATGAAGAAGGTGGAAGTCTCCCGGCCTTACCAGAGCCTGCTGCTGCCCAACCACCGGAAGTTCAAGTACCTGGACGAGACATGGAACGCCTACAAGAGCGTGAAGTCCCTGCTGCACAGATTCCTCGTGTGTGCCTATGGCGCCGTGCCTTTCAACAAGTTCGTGGAAGTGGTGGAAAAGGTGGACAACGATCAGCTGGTGCTGGCCTTTGCCGTGCGGCTGTTTAGACTGGTGCCTGTGGAAAGCACCTCCTTCGCCAAAGTGGACAAGGCCAACCTGGCCAAGAGCCTGGCCAATCATCTGCCTGTGGGCACAGCCATTCCAGCCAACGTGCAGAGCTACTTCGACAGCAACTTCGACCCCAAAAAGTACATGTGGATCGACTGCGCCTGGGAAGCCGACAGACTGGCCAGAGAAATGGGCCTGAGCGCCAGCCAGTTTAGCGAGTACGCCACCACAATGCTGTGGGAAGATTGGCTGCCCCTGAACAAGGACGACGTGAACGGCTGGGGATCTGTGTCTGGCCTGTTTGGCGAGGGCAAGAAAGAGGACCGGCAGCAGAAAGTGAAGATGCTGAACAACCTGCTGAACGGCATCAAGAAGAACCCTCCAAAGGACTACACCCAGTACCTGAAGATCCTGCTGAATGCCTTCGACGCCAAGTCTCACAAAGAAGCCGTGAAGAACTACAAGGGCGACAGCACCGGCAGAACCGCCAGCTACCTGTCTGAGAAGTCTGGCGAGATCACCGAGCTGATGCTGGAACAGCTGATGAGCAACATCCAGAGAGACATCGGCGACAAGCAGAAAGAGATCAGCCTGCCTAAGAAAGACGTGGTCAAGAAGTACCTCGAGAGCGAGAGCGGCGTGCCCTACGACCAAAATCTTTGGAGCCAGGCCTACCGGAATGCCGCCAGCAGCATCAAAAAGACCGACACACGGAACTTCAACAGCACCCTGGAAAAGTTCAAGAACGAGGTGGAACTGCGGGGCCTGCTGTCTGAAGGCGACGATGTGGAAATCCTGCGGA

	GCAAGTTCTTCAGCAGCGAGTTCCACAAGACCCCTGATAAGTTCGTGATC<br>AAGCCCGAGCACATCGGCTTCAACAACAAGTACAACGTGGTGGCCGAGC<br>TGTACAAGCTGAAGGCCGAGGCCACCGATTTCGAGAGCGCCTTTGCCAC<br>CGTGAAGGACGAGTTCGAGGAAAAGGGCATCAAGCACCCCATCAAGAAC<br>ATCCTCGAGTACATCTGGAACAACGAGGTGCCCGTCGAGAAGTGGGGAA<br>GAGTGGCCAGATTCAACCAGAGCGAAGAGAAGCTGCTGCGGATCAAGGC<br>CAATCCTACCGTGGAATGCAACCAGGGCATGACCTTCGGCAACAGCGCC<br>ATGGTTGGAGAGGTGCTGAGAAGCAACTACGTGTCCAAGAAAGGCGCCC<br>TGGTGTCCGGCGAACATGGCGGTAGACTGATCGGCCAGAACAACATGAT<br>CTGGCTGGAAATGCGGCTGCTCAACAAAGGCAAGTGGGAGACACACCAC<br>GTGCCAACACACAACATGAAGTTCTTCGAGGAAGTGCACGCTTACAACCC<br>CAGCCTGGCCGATTCTGTGAACGTGCGGAACCGGCTGTACAGAAGCGAG<br>GATTACACACAGCTGCCCAGCTCCATCACCGATGGCCTGAAGGGAAACC<br>CCAAGGCCAAGCTGCTGAAAAGACAGCACTGCGCCCTCAACAATATGAC<br>CGCCAATGTGCTGAACCCCAAGCTGAGCTTCACCATCAACAAGAAGAATG<br>ACGACTACACCGTGATCATCGTGCACTCTGTGGAAGTGTCCAAGCCTCG<br>GAGAGAAGTGCTCGTGGGCGATTACCTCGTCGGCATGGACCAGAATCAG<br>ACCGCCAGCAATACCTACGCCGTGATGCAGGTCGTGAAGCCCAAGTCTA<br>CCGACGCTATCCCCTTCCGGAATATGTGGGTCCGATTCGTGGAAAGCGG<br>CAGCATCGAGAGCAGAACCCTGAACAGCAGAGGCGAGTACGTGGACCA<br>GCTGAACCATGATGGCGTGGACCTGTTCGAGATCGGCGATACAGAGTGG<br>GTCGACAGCGCCCGGAAATTCTTCAACAAGCTGGGCGTGAAGCACAAGG<br>ACGGCACCCTGGTGGATCTGAGCACAGCCCCTAGAAAGGCCTACGCCTT<br>CAACAATTTCTACTTCAAGACCATGCTCAACCACCTCCGGTCCAACGAAG<br>TGGACCTGACACTGCTGAGAAACGAGATCCTGCGCGTGGCCAACGGCA<br>GATTCAGCCCTATGAGACTGGGCAGCCTGAGCTGGACCACACTGAAAGC<br>CCTGGGGAGCTTCAAGTCCCTGGTGCTGTCCTACTTCGATCGGCTGGGC<br>GCCAAAGAGATGGTCGACAAAGAGGCCAAGGATAAGAGCCTGTTCGACC<br>TGCTGGTGGCCATTAACAACAAGCGGAGCAACAAGAGAGAGGAACGGAC<br>CTCCAGAATCGCCTCCAGCCTGATGACAGTGGCCCAGAAGTATAAGGTCG<br>ACAACGCCGTGGTGCATGTGGTGGTGGAAGGCAATCTGAGCAGCACCGA<br>TCGGAGCGCCTCTAAGGCCCACAACAGAAACACCATGGACTGGTGCAGC<br>AGAGCCGTCGTGAAGAAACTGGAAGATATGTGCAACCTGTACGGGTTCAA<br>CATCAAGGGCGTGCCCGCCTTCTACACCAGCCATCAGGATCCTCTGGTG<br>CACAGGGCCGACTACGACGATCCTAAGCCTGCTCTGCGGTGCCGGTACA<br>GCAGCTACTCTAGAGCCGACTTCTCTAAGTGGGGCCAGAATGCCCTGGC<br>CGCCGTTGTTAGATGGGCCAGCAACAAAAAGTCCAATACCTGCTACAAAG<br>TGGGCGCCGTGGAATTCCTGAAGCAGCACGGACTGTTCGCCGACAAGAA<br>ACTGACCGTGGAACAGTTCCTGAGCAAAGTCAAGGACGAGGAAATTCTG<br>ATCCCTAGAAGAGGCGGCAGAGTGTTCCTGACCACACACAGACTGCTGG<br>CCGAGAGCACCTTCGTGTACCTGAACGGCGTGAAGTACCACAGCTGCAA<br>CGCCGATGAAGTGGCCGCTGTGAATATCTGCCTGAACGACTGGGTCATC<br>CCCTGCAAGAAAAAGATGAAGGAAGAGTCCAGCGCCAGCGGC
Cas12i.3_	ATGAAGAAGGTGGAGGTGAGCCGCCCCTACCAGAGCCTGCTGCTGCCCA

coding sequence _Jcat	ACCACCGCAAGTTCAAGTACCTGGACGAGACCTGGAACGCCTACAAGAG<br>CGTGAAGAGCCTGCTGCACCGCTTCCTGGTGTGCGCCTACGGCGCCGT<br>GCCCTTCAACAAGTTCGTGGAGGTGGTGGAGAAGGTGGACAACGACCA<br>GCTGGTGCTGGCCTTCGCCGTGCGCCTGTTCCGCCTGGTGCCCGTGGA<br>GAGCACCAGCTTCGCCAAGGTGGACAAGGCCAACCTGGCCAAGAGCCT<br>GGCCAACCACCTGCCCGTGGGCACCGCCATCCCCGCCAACGTGCAGAG<br>CTACTTCGACAGCAACTTCGACCCCAAGAAGTACATGTGGATCGACTGCG<br>CCTGGGAGGCCGACCGCCTGGCCCGCGAGATGGGCCTGAGCGCCAGC<br>CAGTTCAGCGAGTACGCCACCACCATGCTGTGGGAGGACTGGCTGCCCC<br>TGAACAAGGACGACGTGAACGGCTGGGGCAGCGTGAGCGGCCTGTTCG<br>GCGAGGGCAAGAAGGAGGACCGCCAGCAGAAGGTGAAGATGCTGAACA<br>ACCTGCTGAACGGCATCAAGAAGAACCCCCCCAAGGACTACACCCAGTA<br>CCTGAAGATCCTGCTGAACGCCTTCGACGCCAAGAGCCACAAGGAGGCC<br>GTGAAGAACTACAAGGGCGACAGCACCGGCCGCACCGCCAGCTACCTG<br>AGCGAGAAGAGCGGCGAGATCACCGAGCTGATGCTGGAGCAGCTGATG<br>AGCAACATCCAGCGCGACATCGGCGACAAGCAGAAGGAGATCAGCCTGC<br>CCAAGAAGGACGTGGTGAAGAAGTACCTGGAGAGCGAGAGCGGCGTGC<br>CCTACGACCAGAACCTGTGGAGCCAGGCCTACCGCAACGCCGCCAGCA<br>GCATCAAGAAAACCGACACCCGCAACTTCAACAGCACCCTGGAGAAGTT<br>CAAGAACGAGGTGGAGCTGCGCGGCCTGCTGAGCGAGGGCGACGACGT<br>GGAGATCCTGCGCAGCAAGTTCTTCAGCAGCGAGTTCCACAAGACCCCC<br>GACAAGTTCGTGATCAAGCCCGAGCACATCGGCTTCAACAACAAGTACAA<br>CGTGGTGGCCGAGCTGTACAAGCTGAAGGCCGAGGCCACCGACTTCGA<br>GAGCGCCTTCGCCACCGTGAAGGACGAGTTCGAGGAGAAGGGCATCAA<br>GCACCCCATCAAGAACATCCTGGAGTACATCTGGAACAACGAGGTGCCC<br>GTGGAGAAGTGGGGCCGCGTGGCCCGCTTCAACCAGAGCGAGGAGAAG<br>CTGCTGCGCATCAAGGCCAACCCCACCGTGGAGTGCAACCAGGGCATGA<br>CCTTCGGCAACAGCGCCATGGTGGGCGAGGTGCTGCGCAGCAACTACG<br>TGAGCAAGAAGGGCGCCCTGGTGAGCGGCGAGCACGGCGGCCGCCTG<br>ATCGGCCAGAACAACATGATCTGGCTGGAGATGCGCCTGCTGAACAAGG<br>GCAAGTGGGAGACCCACCACGTGCCCACCCACAACATGAAGTTCTTCGA<br>GGAGGTGCACGCCTACAACCCCAGCCTGGCCGACAGCGTGAACGTGCG<br>CAACCGCCTGTACCGCAGCGAGGACTACACCCAGCTGCCCAGCAGCATC<br>ACCGACGGCCTGAAGGGCAACCCCAAGGCCAAGCTGCTGAAGCGCCAG<br>CACTGCGCCCTGAACAACATGACCGCCAACGTGCTGAACCCCAAGCTGA<br>GCTTCACCATCAACAAGAAGAACGACGACTACACCGTGATCATCGTGCAC<br>AGCGTGGAGGTGAGCAAGCCCCGCCGCGAGGTGCTGGTGGGCGACTAC<br>CTGGTGGGCATGGACCAGAACCAGACCGCCAGCAACACCTACGCCGTGA<br>TGCAGGTGGTGAAGCCCAAGAGCACCGACGCCATCCCCTTCCGCAACAT<br>GTGGGTGCGCTTCGTGGAGAGCGGCAGCATCGAGAGCCGCACCCTGAA<br>CAGCCGCGGCGAGTACGTGGACCAGCTGAACCACGACGGCGTGGACCT<br>GTTCGAGATCGGCGACACCGAGTGGGTGGACAGCGCCCGCAAGTTCTT<br>CAACAAGCTGGGCGTGAAGCACAAGGACGGCACCCTGGTGGACCTGAG<br>CACCGCCCCCCGCAAGGCCTACGCCTTCAACAACTTCTACTTCAAGACCA

	TGCTGAACCACCTGCGCAGCAACGAGGTGGACCTGACCCTGCTGCGCA<br>ACGAGATCCTGCGCGTGGCCAACGGCCGCTTCAGCCCCATGCGCCTGG<br>GCAGCCTGAGCTGGACCACCCTGAAGGCCCTGGGCAGCTTCAAGAGCC<br>TGGTGCTGAGCTACTTCGACCGCCTGGGCGCCAAGGAGATGGTGGACAA<br>GGAGGCCAAGGACAAGAGCCTGTTCGACCTGCTGGTGGCCATCAACAAC<br>AAGCGCAGCAACAAGCGCGAGGAGCGCACCAGCCGCATCGCCAGCAGC<br>CTGATGACCGTGGCCCAGAAGTACAAGGTGGACAACGCCGTGGTGCACG<br>TGGTGGTGGAGGGCAACCTGAGCAGCACCGACCGCAGCGCCAGCAAGG<br>CCCACAACCGCAACACCATGGACTGGTGCAGCCGCGCCGTGGTGAAGA<br>AGCTGGAGGACATGTGCAACCTGTACGGCTTCAACATCAAGGGCGTGCC<br>CGCCTTCTACACCAGCCACCAGGACCCCCTGGTGCACCGCGCCGACTAC<br>GACGACCCCAAGCCCGCCCTGCGCTGCCGCTACAGCAGCTACAGCCGC<br>GCCGACTTCAGCAAGTGGGGCCAGAACGCCCTGGCCGCCGTGGTGCGC<br>TGGGCCAGCAACAAGAAGAGCAACACCTGCTACAAGGTGGGCGCCGTG<br>GAGTTCCTGAAGCAGCACGGCCTGTTCGCCGACAAGAAGCTGACCGTG<br>GAGCAGTTCCTGAGCAAGGTGAAGGACGAGGAGATCCTGATCCCCCGCC<br>GCGGCGGCCGCGTGTTCCTGACCACCCACCGCCTGCTGGCCGAGAGCA<br>CCTTCGTGTACCTGAACGGCGTGAAGTACCACAGCTGCAACGCCGACGA<br>GGTGGCCGCCGTGAACATCTGCCTGAACGACTGGGTGATCCCCTGCAAG<br>AAGAAGATGAAGGAGGAGAGCAGCGCCAGCGGC
Cas12i.3_ coding sequence _General Biol	ATGAAGAAGGTGGAGGTGAGCAGGCCTTACCAGTCCCTGCTGCTGCCTA<br>ACCACAGGAAGTTTAAGTACCTGGATGAGACATGGAACGCCTACAAGTCC<br>GTGAAGAGCCTGCTGCACAGGTTTCTGGTGTGCGCTTACGGCGCTGTGC<br>CTTTTAACAAGTTCGTGGAGGTGGTGGAGAAGGTGGACAACGACCAGCT<br>GGTGCTGGCTTTTGCCGTGAGACTGTTCAGGCTGGTGCCTGTTGAGAGC<br>ACATCCTTTGCCAAGGTGGATAAGGCCAACCTGGCCAAGTCCCTCGCTAA<br>CCATCTGCCTGTGGGCACAGCTATCCCCGCTAATGTGCAGAGCTACTTTG<br>ACTCCAATTTCGATCCCAAGAAGTATATGTGGATCGACTGCGCCTGGGAG<br>GCCGACAGGCTGGCTAGGGAGATGGGACTGTCTGCTTCCCAGTTTAGCG<br>AGTACGCCACAACAATGCTGTGGGAGGACTGGCTGCCCCTGAATAAGGA<br>CGATGTGAATGGCTGGGGCTCCGTGTCTGGACTGTTCGGAGAAGGAAAG<br>AAGGAGGACAGACAGCAGAAGGTGAAGATGCTGAATAACCTGCTGAATG<br>GCATCAAGAAGAACCCCCCCAAGGACTACACACAGTACCTGAAGATCCTG<br>CTGAACGCCTTCGATGCCAAGTCCCACAAGGAGGCTGTGAAGAACTACA<br>AGGGCGACTCCACCGGCAGAACAGCTTCTTACCTGTCCGAGAAGTCCGG<br>CGAGATCACCGAACTGATGCTGGAGCAGCTGATGTCCAACATCCAGAGG<br>GACATCGGCGACAAGCAGAAGGAGATCTCCCTGCCTAAGAAGGATGTGG<br>TGAAGAAGTACCTGGAGAGCGAGTCCGGCGTGCCTTATGATCAGAACCT<br>GTGGAGCCAGGCCTACAGGAATGCCGCTTCTAGCATCAAGAAAACTGATA<br>CTAGGAACTTCAACAGCACCCTGGAGAAGTTTAAGAATGAGGTGGAGCTG<br>AGAGGCCTGCTGTCCGAGGGAGATGATGTGGAAATCCTGAGGTCCAAGT<br>TCTTTAGCTCCGAGTTTCACAAGACACCTGATAAGTTTGTGATCAAGCCTG<br>AGCACATCGGCTTTAATAACAAGTACAACGTGGTGGCCGAGCTGTATAAAC<br>TGAAGGCCGAGGCCACAGATTTTGAGAGCGCCTTCGCCACAGTGAAGGA

	TGAGTTTGAGGAGAAGGGCATCAAGCACCCCATCAAGAACATCCTGGAG<br>TACATCTGGAACAACGAGGTGCCTGTGGAGAAGTGGGGCAGAGTTGCTA<br>GATTCAACCAGTCCGAGGAGAAGCTGCTGAGGATCAAGGCCAATCCTAC<br>AGTGGAGTGCAATCAGGGCATGACATTCGGCAACTCCGCCATGGTGGGC<br>GAAGTTCTGAGGTCTAATTACGTGAGCAAGAAGGGCGCCCTGGTGTCTG<br>GAGAACACGGAGGAAGATTGATTGGTCAGAACAACATGATCTGGCTGGA<br>GATGAGACTGCTGAATAAGGGCAAGTGGGAGACACACCACGTGCCAACA<br>CACAACATGAAGTTCTTCGAGGAGGTGCACGCTTACAACCCTTCCCTGGC<br>TGACTCCGTGAATGTGAGGAACAGGCTGTACCGGAGCGAGGATTACACA<br>CAGCTGCCCTCTTCCATCACAGACGGCCTGAAGGGAAATCCCAAGGCCA<br>AGCTGCTGAAGAGGCAGCATTGCGCTCTGAACAACATGACCGCCAACGT<br>GCTGAATCCCAAGCTGTCCTTCACAATCAACAAGAAGAACGACGACTACA<br>CCGTGATCATCGTGCACTCCGTGGAGGTGTCCAAGCCAAGAAGAGAGGT<br>GCTGGTGGGCGATTACCTGGTGGGAATGGACCAGAATCAGACCGCCTCC<br>AATACCTACGCCGTGATGCAGGTGGTGAAGCCTAAGAGCACCGATGCCAT<br>CCCCTTCAGAAATATGTGGGTGAGATTTGTGGAGAGCGGCAGCATCGAGA<br>GCAGAACACTGAACAGCAGGGGCGAGTACGTGGATCAGCTGAACCACGA<br>TGGCGTGGATCTGTTTGAGATCGGCGACACAGAGTGGGTGGACTCTGCT<br>AGAAAGTTCTTCAATAAGCTGGGCGTGAAGCACAAGGATGGCACCCTGG<br>TTGACCTGAGCACAGCTCCTAGAAAGGCCTACGCCTTTAACAACTTTTACT<br>TCAAGACCATGCTGAACCACCTGAGGAGCAATGAGGTGGATCTGACCCT<br>GCTGAGGAACGAGATCCTGAGAGTGGCCAATGGCAGGTTCTCCCCAATG<br>AGACTGGGCTCTCTGTCCTGGACAACCCTGAAGGCTCTGGGATCTTTTAA<br>GTCCCTGGTGCTGAGCTACTTCGATAGGCTGGGCGCTAAGGAGATGGTG<br>GATAAGGAGGCCAAGGACAAGTCCCTGTTCGATCTGCTGGTGGCCATCA<br>ATAACAAGAGATCCAATAAGAGGGAGGAGAGAACATCCAGAATCGCCTCC<br>AGCCTGATGACCGTGGCTCAAAAGTACAAGGTGGACAATGCCGTGGTGC<br>ACGTGGTGGTTGAGGGAAATCTGAGCAGCACAGATAGATCCGCCAGCAA<br>GGCCCACAATAGGAATACCATGGACTGGTGTTCCAGAGCCGTCGTTAAGA<br>AGCTGGAGGATATGTGTAACCTGTACGGCTTTAACATCAAGGGCGTGCCT<br>GCCTTTTACACAAGCCATCAAGATCCTCTGGTGCACAGAGCCGACTACGA<br>CGATCCTAAGCCCGCTCTGAGGTGCAGATACAGCAGCTACAGCAGAGCC<br>GACTTCTCCAAGTGGGGCCAAAACGCTCTGGCCGCTGTTGTTAGATGGG<br>CCTCTAATAAGAAGTCCAACACATGCTACAAGGTGGGCGCCGTGGAGTTT<br>CTGAAGCAGCATGGACTGTTTGCCGACAAGAAGCTGACAGTGGAGCAGT<br>TCCTGTCCAAGGTGAAGGATGAGGAGATCCTGATCCCTAGAAGGGGCGG<br>AAGGGTGTTTCTGACAACCCACAGACTGCTGGCCGAGTCTACCTTCGTG<br>TACCTGAACGGCGTGAAGTACCACTCCTGTAATGCCGATGAGGTGGCCG<br>CTGTGAACATCTGTCTGAATGACTGGGTCATTCCCTGCAAGAAGAAGATG<br>AAGGAGGAGTCCAGCGCCTCCGGA
T5 exonuclease	MSKSWGKFIEEEEAEMASRRNLMIVDGTNLGFRFKHNNSKKPFASSYVSTIQ<br>SLAKSYSARTTIVLGDKGKSVFRLEHLPEYKGNRDEKYAQRTEEEKALDEQF<br>FEYLKDAFELCKTTFPTFTIRGVEADDMAAYIVKLIGHLYDHVWLISTDGDWD<br>TLLTDKVSRFSFTTRREYHLRDMYEHHNVDDVEQFISLKAIMGDLGDNIRGV

	EGIGAKRGYNIIREFGNVLDIIDQLPLPGKQKYIQNLNASEELLFRNLILVDLPT YCVDAIAAVGQDVLDKFTKDILEIAEQ
T5 exonuclease_coding sequence	ATGAGCAAGAGCTGGGGCAAGTTCATCGAGGAAGAGGAGGCCGAGATG GCTAGCAGAAGAAACCTGATGATCGTGGACGGCACCAACCTGGGCTTCA GATTCAAGCACAACAACAGCAAGAAGCCCTTCGCTAGCAGCTACGTGAG CACCATTCAGAGCCTGGCCAAGAGCTACAGCGCTAGAACCACCATCGTG CTGGGCGACAAGGGCAAGAGCGTGTTCAGACTGGAGCACCTGCCCGAG TACAAGGGCAACAGAGACGAGAAGTACGCTCAGAGAACCGAGGAAGAGA AGGCCCTGGACGAGCAGTTCTTCGAGTACCTGAAGGACGCCTTCGAGCT GTGCAAGACCACCTTCCCCACCTTCACCATTAGAGGCGTGGAGGCTGAC GACATGGCCGCCTACATCGTGAAGCTGATCGGCCACCTGTACGACCACG TGTGGCTGATCAGCACCGACGGCGACTGGGACACCCTGCTGACCGACA AGGTGAGCAGATTCAGCTTCACCACAAGAAGAGAGTACCACCTGAGAGA CATGTACGAGCACCACAACGTGGACGACGTGGAGCAGTTCATCAGCCTG AAGGCCATCATGGGCGACCTGGGCGACAACATCCGGGGCGTGGAAGGG ATTGGCGCCAAGAGAGGCTACAACATCATCAGAGAGTTCGGCAACGTGC TGGATATCATCGATCAGCTGCCCCTGCCCGGCAAGCAGAAGTACATTCAG AACCTGAACGCTAGCGAGGAGCTGCTGTTCAGAAACCTGATCCTGGTGG ACCTGCCCACCTACTGCGTGGACGCCATCGCCGCCGTGGGCCAAGACG TGCTGGACAAGTTCACCAAGGACATCCTGGAGATCGCCGAGCAG
IOCas12i protein	MKKVEVSRPYQSLLLPNHRKFKYLDETWNAYKSVKSLLHRFLVCAYGAVPFN KFVEVVEKVDNDQLVLAFAVRLFRLVPVESTSFAKVDKANLAKSLANHLPVGT AIPANVQSYFDSNFDPKKYMWIDCAWEADRLAREMGLSASQFSEYATTMLW EDWLPLNKDDVNGWGSVSGLFGEGKKEDRQQKVKMLNNLLNGIKKNPPKD YTQYLKILLNAFDAKSHKEAVKNYKGDSTGRTASYLSEKSGEITELMLEQLMS NIQRDIGDKQKEIRLPKKDVVKKYLESESGVPYDQNLWSQAYRNAASSIKKT DTRNFNSTLEKFKNEVELRGLLSEGDDVEILRSKFFSSEFHKTPDKFVIKPEHI GFNNKYNVVAELYKLKAEATDFESAFATVKDEFEEKGIKHPIKNILEYIWNNEV PVEKWGRVARFNQSEEKLLRIKANPTVECNQGMTFGNSAMVGEVLRSNYV SKKGALVSGEHGGRLIGQNNMIWLEMRLLNKGKWETHHVPTHNMKFFEEV HAYNPSLADSVNVRNRLYRSEDYTQLPSSITDGLKGNPKAKLLKRQHCALNN MTANVLNPKLSFTINKKNDDYTVIIVHSVEVSKPRREVLVGDYLVGMDQNQTA SNTYAVMQVVKPKSTDAIPFRNMWVRFVESGSIESRTLNSRGEYVDQLNHD GVDLFEIGDTEWVDSARKFFNKLGVKHKDGTLVDLSTAPRKAYAFNNFYFKT MLNHLRSNEVDLTLLRNEILRVANGRFSPMRLGSLSWTTLKALGSFKSLVLSY FDRLGAKEMVDKEAKDKSLFDLLVAINNKRSNKREERTSRIASSLMTVAQKY KVDNAVVHVVVEGNLSSTDRSASKAHNRNTMDWCSRAVVKKLEDMCNLYG FNIKGVPAFYTSHQDPLVHRADYDDPKPALRCRYSSYSRADFSKWGQNALA AVVRWASNKKSNTCYKVGAVEFLKQHGLFADKKLTVEQFLSKVKDEEILIPRR GGRVFLTTHRLLAESTFVYLNGVKYHSCNADEVAAVNICLNDWVIPCKKKMK EESSASGSGGSGGSGGSMSKSWGKFIEEEEAEMASRRNLMIVDGTNLGFR FKHNNSKKPFASSYVSTIQSLAKSYSARTTIVLGDKGKSVFRLEHLPEYKGNR DEKYAQRTEEEKALDEQFFEYLKDAFELCKTTFPTFTIRGVEADDMAAYIVKLI GHLYDHVWLISTDGDWDTLLTDKVSRFSFTTRREYHLRDMYEHHNVDDVEQ

	FISLKAIMGDLGDNIRGVEGIGAKRGYNIIREFGNVLDIIDQLPLPGKQKYIQNL<br>NASEELLFRNLILVDLPTYCVDAIAAVGQDVLDKFTKDILEIAEQ
EOCas12i<br>-Combo1<br>protein	MKKVEVSRPYQSLLLPNHRKFKYLDETWNAYKSVKSLLHRFLVCAYGAVPFN<br>KFVEVVEKVDNDQLVLAFAVRLFRLVPVESTSFAKVDKANLAKSLANHLPVGT<br>AIPANVQSYFDSNFDPKKYMWIDCAWEADRLAREMGLSASQFSEYATTMLW<br>EDWLPLNKDDVRGWGSVSGLFGEGKKEDRQQKVMLNNLLNGIKKNPPKD<br>YTQYLKILLNAFDAKSHKEAVKNYKGDSTGRTASYLSEKSGEITELMLEQLMS<br>NIQRDIGDKQKEIRLPKKDVVKKYLESESGVPYDQNLWSQAYRNAASSIKKT<br>DTRNFNSTLEKFKNEVELRGRLSEGDDVEILRSKFFSSEFHKTPDKFVIKPEH<br>IGFNNKYNVVAELYKLKAEATDFESAFATVKDEFEEKGIKHPIKNILEYIWNNEV<br>PVEKWGRVARFNQSEEKLLRIKANPTVECNQGMTFGNSAMVGEVLRSNYV<br>SKKGALVSREHGGRLIGQNNMIWLEMRLLNKGKWETHHVPTHNMKFFEEV<br>HAYNPSLADSVNVRNRLYRSEDYTQLPSSITDGLKGNPKAKLLKRQHCALNN<br>MTANVLNPKLSFTINKKNDDYTVIIVHRVEVSKPRREVLVGDYLVGMDQNQTA<br>SNTYAVMQVVKPKSTDAIPFRNMWVRFVESGSIESRTLNSRGEYVDQLNHD<br>GVDLFEIGDTEWVDSARKFFNKLGVKHKDGTLVDLSTAPRKAYAFNNFYFKT<br>MLNHLRSNEVDLTLLRNEILRVANGRFSPMRLGSLSWTTLKALGSFKSLVLSY<br>FDRLGAKEMVDKEAKDKSLFDLLVAINNKRSNKREERTSRIASSLMTVAQKY<br>KVDNAVVHVVVEGNLSSTDRSASKAHNRNTMDWCSRAVVKKLEDMCNLYG<br>FNIKGVPAFYTSHQDPLVHRADYDDPKPALRCRYSSYSRADFSKWGQNALA<br>AVVRWASNKKSNTCYKVGAVEFLKQHGLFADKKLTVEQFLSKVKDEEILIPRR<br>GGRVFLTTHRLLAESTFVYLNGVKYHSCNADEVAAVNICLNDWVIPCKKKMK<br>EESSASGSGGSGGSGGSMSKSWGKFIEEEEAEMASRRNLMIVDGTNLGFR<br>FKHNNSKKPFASSYVSTIQSLAKSYSARTTIVLGDKGKSVFRLEHLPEYKGNR<br>DEKYAQRTEEEKALDEQFFEYLKDAFELCKTTFPTFTIRGVEADDMAAYIVKLI<br>GHLYDHVWLISTDGDWDTLLTDKVSRFSFTTRREYHLRDMYEHHNVDDVEQ<br>FISLKAIMGDLGDNIRGVEGIGAKRGYNIIREFGNVLDIIDQLPLPGKQKYIQNL<br>NASEELLFRNLILVDLPTYCVDAIAAVGQDVLDKFTKDILEIAEQ
EOCas12i<br>-Combo2<br>protein	MKKVEVRRPYQSLLLPNHRKFKYLDETWNAYKSVKSLLHRFLVCAYGAVPFN<br>KFVEVVEKVDNDQLVLAFAVRLFRLVPVESTSFAKVDKANLAKSLANHLPVGT<br>AIPANVQSYFDSNFDPKKYMWIDCAWEADRLAREMGLSASQFSEYATTMLW<br>EDWLPLNKDDVRGWGSVSGLFGEGKKEDRQQKVMLNNLLNGIKKNPPKD<br>YTQYLKILLNAFDAKSHKEAVKNYKGDSTGRTASYLSEKSGEITELMLEQLMS<br>NIQRDIGDKQKEIRLPKKDVVKKYLESESGVPYDQNLWSQAYRNAASSIKKT<br>DTRNFNSTLEKFKNEVELRGRLSEGDDVEILRSKFFSSEFHKTPDKFVIKPEH<br>IGFNNKYNVVAELYKLKAEATDFESAFATVKDEFEEKGIKHPIKNILEYIWNNEV<br>PVEKWGRVARFNQSEEKLLRIKANPTVECNQGMTFGNSAMVGEVLRSNYV<br>SKKGALVSREHGGRLIGQNNMIWLEMRLLNKGKWERHHVPTHNMKFFEEV<br>HAYNPSLADSVNVRNRLYRSEDYTQLPSSITRGLKGNPKAKLLKRQHCALNN<br>MTANVLNPKLSFTINKKNDDYTVIIVHSVEVSKPRREVLVGDYLVGMDQNQTA<br>SNTYAVMQVVKPKSTDAIPFRNMWVRFVESGSIESRTLNSRGEYVDQLNHD<br>GVDLFEIGDTEWVDSARKFFNKLGVKHKDGTLVDLSTAPRKAYAFNNFYFKT<br>MLNHLRSNEVDLTLLRNEILRVANGRFSPMRLGSLSWTTLKALGSFKSLVLSY<br>FDRLGAKEMVDKEAKDKSLFDLLVAINNKRSNKREERTSRIASSLMTVAQKY

	KVDNAVVHVVVEGNLSSTDRSASKAHNRNTMDWCSRAVVKKLEDMCNLYG FNIKGVPAFYTSHQDPLVHRADYDDPKPALRCRYSSYSRADFSKWGQNALA AVVRWASNKKSNTCYKVGAVEFLKQHGLFADKKLTVEQFLSKVKDEEILIPRR GGRVFLTTHRLLAESTFVYLNGVKYHSCNADEVAAVNICLNDWVIPCKKKMK EESSASGSGGSGGSGGSMSKSWGKFIEEEEAEMASRRNLMIVDGTNLGFR FKHNNSKKPFASSYVSTIQSLAKSYSARTTIVLGDKGKSVFRLEHLPEYKGNR DEKYAQRTEEEKALDEQFFEYLKDAFELCKTTFPTFTIRGVEADDMAAYIVKLI GHLYDHVWLISTDGDWDTLLTDKVSRFSFTTRREYHLRDMYEHHNVDDVEQ FISLKAIMGDLGDNIRGVEGIGAKRGYNIIREFGNVLDIIDQLPLPGKQKYIQNL NASEELLFRNLILVDLPTYCVDAIAAVGQDVLDKFTKDILEIAEQ
full Direct-repeat	CUCUGACCACCUGAGAGAAUGUGUGCAUAGUCACAC
truncated Direct-repeat	AGAGAAUGUGUGCAUAGUCACAC
full Direct-repeat (DNA)	CTCTGACCACCTGAGAGAATGTGTGCATAGTCACAC
truncated Direct-repeat (DNA)	AGAGAATGTGTGCATAGTCACAC
DRf-1	CUCUGACCACCUGAGAGAAU**GGGUG**CAUAGU**CACCC**
DRf-2	CUCUGACCACCUGAGAGAAU**GCGUG**CAUAGU**CACGC**
DRf-3	CUCUGACCACCUGAGAGAAU**GUGGG**CAUAGU**CCCAC**
DRf-4	CUCUGACCACCUGAGAGAAU**GUGCG**CAUAGU**CGCAC**
DRf-13	CUCUGACCACCUGAGAGAAU**GGGGG**CAUAGU**CCCCC**
DRf-14	CUCUGACCACCUGAGAGAAU**GGGCG**CAUAGU**CGCCC**
DRf-23	CUCUGACCACCUGAGAGAAU**GCGGG**CAUAGU**CCCGC**
DRf-24	CUCUGACCACCUGAGAGAAU**GCGCG**CAUAGU**CGCGC**
DRf-1 (DNA)	CTCTGACCACCTGAGAGAATGGGTGCATAGTCACCC
DRf-2 (DNA)	CTCTGACCACCTGAGAGAATGCGTGCATAGTCACGC
DRf-3 (DNA)	CTCTGACCACCTGAGAGAATGTGGGCATAGTCCCAC
DRf-4 (DNA)	CTCTGACCACCTGAGAGAATGTGCGCATAGTCGCAC
DRf-13 (DNA)	CTCTGACCACCTGAGAGAATGGGGGCATAGTCCCCC
DRf-14 (DNA)	CTCTGACCACCTGAGAGAATGGGCGCATAGTCGCCC
DRf-23 (DNA)	CTCTGACCACCTGAGAGAATGCGGGCATAGTCCCGC

DRf-24 (DNA)	CTCTGACCACCTGAGAGAATGCGCGCATAGTCGCGC
Linker 1	GGGGSGGGGS (10 aa)
Linker 2	GGGGSGGGGSGGGGS (15 aa)
Linker 3	SGGSGGSGGS (10 aa)
Linker 4	SGSETPGTSESATPES (16 aa)
Linker 1_coding sequence	GGAGGCGGAGGATCTGGCGGAGGCGGATCT
Linker 2_coding sequence	GGAGGCGGAGGATCTGGCGGAGGTGGAAGTGGCGGAGGCGGATCT
Linker 3_coding sequence	TCTGGTGGAAGCGGAGGATCTGGCGGATCT
Linker 4_coding sequence	AGCGGATCTGAGACACCTGGCACAAGCGAGTCTGCCACACCTGAGTCT
Triplex sequence	GATTCGTCAGTAGGGTTGTAAAGGTTTTTCTTTTCCTGAGAAAACAACCTTTTGTTTTCTCAGGTTTTGCTTTTTGGCCTTTCCCTAGCTTTAAAAAAAAAAAAGCAAAA
20-target_array	AGAGAATGTGcGCATAGTCgCACCGGATGTCCACGTCACACTTCATAGAGAATGTGcGCATAGTCgCACCCTCTCAGGCATGGAGTCCTGTGAGAGAATGTGcGCATAGTCgCACGCTACAGCAACAGGGTGGTGGACAGAGAATGTGcGCATAGTCgCACAACAGCGACACCCACTCCTCCACAGAGAATGTGcGCATAGTCgCACGAGCCCCCAGAACTGCAGCATCAAGAGAATGTGcGCATAGTCgCACAGGGTGAACTTTGGTGGGAACCGAGAGAATGTGcGCATAGTCgCACCTCTAGGAACCCTCAGCCCCAAGAGAGAATGTGcGCATAGTCgCACTGCCTGTATGTGCGTGCCTCGGTAGAGAATGTGcGCATAGTCgCACGCACATCTTCTTGGCCTTCGGTCAGAGAATGTGcGCATAGTCgCACGTAAAATCCAGATCCTGCACAAGAGAGAATGTGcGCATAGTCgCACTTCACTGACAAAGACAGTAAATTAGAGAATGTGcGCATAGTCgCACCCTATATTTCTATGTGGTCTTGTAGAGAATGTGcGCATAGTCgCACTCTCCCCTTCCTCCCCAGACACGAGAGAATGTGcGCATAGTCgCACCATGTCTCCCCCCATGAGGCAGGAGAGAATGTGcGCATAGTCgCACGCAAGGATGGACGCGCCACAGAGAGAGAATGTGcGCATAGTCgCACAGGGAAGCGGGATGCGCCTGAAGAGAGAATGTGcGCATAGTCgCACAAGTCCAACTCCTAAGCCAGTGCAGAGAATGTGcGCATAGTCgCACGTAGCAATTTGTACTGATGGTATAGAGAATGTGcGCATAGTCgCACGGTAAGCTCCTTCCACTCTCATTAGAGAATGTGcGCATAGTCgCACCAGGAGGGTGACTCAGGCTAGCAAGAGAATGTGcGCATAGTCgCAC
30-target_array	AGAGAATGTGcGCATAGTCgCACCGGATGTCCACGTCACACTTCATAGAGAATGTGcGCATAGTCgCACCCTCTCAGGCATGGAGTCCTGTGAGAGAATGTGcGCATAGTCgCACGCTACAGCAACAGGGTGGTGGACAGAGAATGTGcGCATAGTCgCACAACAGCGACACCCACTCCTCCACAGAGAATGTGcGCATAG

	TCgCACGAGCCCCCAGAACTGCAGCATCAAGAGAATGTGcGCATAGTCg CACAGGGTGAACTTTGGTGGGAACCGAGAGAATGTGcGCATAGTCgCACC TCTAGGAACCCTCAGCCCCAAGAGAGAATGTGcGCATAGTCgCACTGCCT GTATGTGCGTGCCTCGGTAGAGAATGTGcGCATAGTCgCACGCACATCTTC TTGGCCTTCGGTCAGAGAATGTGcGCATAGTCgCACGTAAAATCCAGATCC TGCACAAGAGAGAATGTGcGCATAGTCgCACTTCACTGACAAAGACAGTAA ATTAGAGAATGTGcGCATAGTCgCACCCTATATTTCTATGTGGTCTTGTAGA GAATGTGcGCATAGTCgCACTCTCCCCTTCCTCCCCAGACACGAGAGAAT GTGcGCATAGTCgCACCATGTCTCCCCCCATGAGGCAGGAGAGAATGTGc GCATAGTCgCACGCAAGGATGGACGCGCCACAGAGAGAGAATGTGcGCAT AGTCgCACAGGGAAGCGGGATGCGCCTGAAGAGAGAATGTGcGCATAGT CgCACAAGTCCAACTCCTAAGCCAGTGCAGAGAATGTGcGCATAGTCgCA CGTAGCAATTTGTACTGATGGTATAGAGAATGTGcGCATAGTCgCACGGTAA GCTCCTTCCACTCTCATTAGAGAATGTGcGCATAGTCgCACCAGGAGGGT GACTCAGGCTAGCAAGAGAATGTGcGCATAGTCgCACGGGTGTTCTGTCA CAGAAGACAAAGAGAATGTGcGCATAGTCgCACTTCTGCAGGAAGCGGTC TAGCAAAGAGAATGTGcGCATAGTCgCACCTGATGGTCCATGTCTGTTACT CAGAGAATGTGcGCATAGTCgCACCCATAGAGTCCTTGGTGGCCAAGAGA GAATGTGcGCATAGTCgCACTGGTTGCCCACCCTAGTCATTGGAGAGAAT GTGcGCATAGTCgCACCCCAGAAGCCAGTGGACTAGCACAGAGAATGTGc GCATAGTCgCACGGTTCTCTCTATAGCCATTGAAGAGAGAATGTGcGCATA GTCgCACGGCGGGGTCCAGTTCCGGGATTAAGAGAATGTGcGCATAGTCg CACGTGCTCAATGAAAGGAGATAAGGAGAGAATGTGcGCATAGTCgCACC TAGGAATATTGAAGGGGGCAGGAGAGAATGTGcGCATAGTCgCAC
HH	NNNNNNCUGAUGAGUCCGUGAGGACGAAACGAGUAAGCUCGUC
HDV	GGCCGGCAUGGUCCCAGCCUCCUCGCUGGCGCCGGCUGGGCAACAUG CUUCGGCAUGGCGAAUGGGAC
HH (DNA)	NNNNNNCTGATGAGTCCGTGAGGACGAAACGAGTAAGCTCGTC
HDV (DNA)	GGCCGGCATGGTCCCAGCCTCCTCGCTGGCGCCGGCTGGGCAACATGC TTCGGCATGGCGAATGGGAC

Table S2

**1.The target sequences of ZFX and the corresponding T7E1 primers.**					
**Targets**	**Guide_sequence**	**PCR_primer_name**	**Sequence (5'- 3')**	**Nuclease**	**PAM**
Sheep_ ZFX	CAGTACAGCAA GAGTGGATGAA T	Sheep_ZFX_T7E1_F	ATGGTGT AGGTAGG GGAGTG	Cas12i.3	TTC
	ATGTTCCAGTAC AGCAAGAG	Sheep_ZFX_T7E1_ R	AATTGGG GAAGTAT CGGATC	SpCas9	TG G
**2.The target sequences of tdTomato in the fluorescence reporter system.**					
**Targets**	**Guide_sequence**				

tdTomato	AAGACCATCTACATGGCCAAGAA		
**3.The target sequences used for screening single mutants and the corresponding T7E1 primers.**			
**Targets**	**Guide_sequence**	**PCR_primer_name**	**Sequence (5'- 3')**
HEK293T_FANCF	GGCGGGGTCCAGTTCCGGGATTA	HEK293T_FANCF_T7E1_F	TGCCCAGAGTCAAGGAACAC
		HEK293T_FANCF_T7E1_R	CCAGGCTCTCTTGGAGTGTC
**4.The target sites and identification primers for gene-edited mice.**			
**Targets**	**Guide_sequence**	**PCR_primer_name**	**Sequence (5'- 3')**
DNMT1	AGAAGGGGCTTTGTAGATGACCT	DNMT1_PAGE_F	GTGGGCGAGTGCGAAAACAT
		DNMT1_PAGE_R	ACATAGGGACGAAAGGAGGA

Table S3

**Cas12i targeting site**	**Guide_sequence**	**PCR_primer_name**	**Sequence (5'- 3')**
HEK293T_EMX1_site 1	CGCAGGACCCAGGGGTAGAAATG	HEK293T_EMX1_site1_T7E1_F	CGGCTTTACCATAGAGTCCT
		HEK293T_EMX1_site1_T7E1_R	CCAGTGGAGGATCTTTATTA
HEK293T_EMX1_site 2	CCATAGAGTCCTTGGTGGCCAAG	HEK293T_EMX1_site2_T7E1_F	CTCTGAGAACTGAAACGACATC
		HEK293T_EMX1_site2_T7E1_R	GGGTAGAAATGGAGAGGGTC
HEK293T_FANCF	CCCAGAAGCCAGTGGACTAGCAC	HEK293T_FANCF_T7E1_F	GGAACACGGATAAAGACGCTG
		HEK293T_FANCF_T7E1_F	AGGGCACATCTTGGGACTCAG
**SpCas9 targeting site**	**Guide_sequence**	**PCR_primer_name**	**Sequence (5'- 3')**
HEK293T_EMX1_site 1	GCAGGACCCAGGGGTAGAAA	HEK293T_EMX1_site1_T7E1_F	CGGCTTTACCATAGAGTCCT
		HEK293T_EMX1_site1_T7E1_R	CCAGTGGAGGATCTTTATTA
HEK293T_	CATAGAGTCCTTGGTG	HEK293T_EMX1_site	CTCTGAGAACTGA

EMX1_site 2	GCCA	2_T7E1_F	AACGACATC
		HEK293T_EMX1_site 2_T7E1_R	GGGTAGAAATGGA GAGGGTC
HEK293T_ FANCF	GAAGCCAGTGGACTA GCACT	HEK293T_FANCF_T7E1_ F	GGAACACGGATAA AGACGCTG
		HEK293T_FANCF_T7E1_ F	AGGGCACATCTTG GGACTCAG
**Targets**	**Guide_sequence**	**PCR_primer_name**	**Sequence (5'- 3')**
NIH- 3T3_DNMT 1_site 1	CCTGGAGGAGGAACA GTTAATGA	NIH-3T3_DNMT1_site 1_T7E1_F	CAGTCCCGTGTTG GTTGTATG
		NIH-3T3_DNMT1_site 1_T7E1_R	TGCTCCCACAAAC CTGAATCT
NIH- 3T3_DNMT 1_site 2	AGAAGGGGCTTTGTA GATGACCT	NIH-3T3_DNMT1_site 2_T7E1_F	CAGGTCGTGTAAG TCGCCAA
		NIH-3T3_DNMT1_site 2_T7E1_R	GCAGCACCACAAA TGAGGAT
NIH- 3T3_EMX1	CCCTAGTCATTGGAG GTGACATC	NIH-3T3_EMX1_T7E1_F	GTTCTTCGTGTAG ACGCCTC
		NIH-3T3_EMX1_T7E1_R	TAGGGAAGGGGG ACATGAGA
NIH- 3T3_FANC F	CGAGAAGCCAGCGCA GTAGTTTC	NIH- 3T3_FANCF_T7E1_F	AACAAAAGAGCAC ATAAAATC
		NIH- 3T3_FANCF_T7E1_R	GTGAACACTAACA TAGGCAGG

Table S4

**Name**	**Targets (5'- 3')**	**PCR_primer_name**	**Sequence (5'- 3')**
Cas12i.3_ TS	CCATAGAGTCCTTGGTGGC CAAG	Cas12i.3_TS_F	GCGGCTGCGACCA TGTTCCA
		Cas12i.3_TS_R	CGGCAGGGAAGCC ACTCACG
Cas12i.3_ OTS 1	CtATAaAGTaCTTGGTGGCaA AG	Cas12i.3_OTS 1_F	TTTTTGAGGTCCAC AATCCC
		Cas12i.3_OTS 1_R	GCAGCATGGCAGA CATAATC
Cas12i.3_ OTS 2	aCATAGAGTCCaTGGaGcCC AAG	Cas12i.3_OTS 2_F	AAGACTAACACCTA CCTCAA
		Cas12i.3_OTS 2_R	GAACCTTCTACACT CTATCC
Cas12i.3_ OTS 3	CtATAGAGTCtTTGGTGGCtA tG	Cas12i.3_OTS 3_F	CACTGTGCCTTTGG TTATGA

		Cas12i.3_OTS 3_R	CCATGCACTGTTCA CTTGAT
Cas12i.3_ OTS 4	CCATAGA- TCCTTGaaGGCCAAa	Cas12i.3_OTS 4_F	TTCTACAAGTGATT GGGTTC
		Cas12i.3_OTS 4_R	ATCTTTCTTCATTCG TTTCC
Cas12i.3_ OTS 5	CCcaAGAGTCCTTGGgGGC C-AG	Cas12i.3_OTS 5_F	CCTACCACCTCTCT AAGCAGC
		Cas12i.3_OTS 5_R	GGACTGAAGGGGG AGAAAACT
SpCas9_ TS	CATAGAGTCCTTGGTGGCC A	SpCas9_TS_F	GCGGCTGCGACCA TGTTCCA
		SpCas9_TS_R	CGGCAGGGAAGCC ACTCACG
SpCas9_ OTS 1	CATAGAGTtCTgGGTGGCtA AGG	SpCas9_OTS 1_F	CCTTATGACCCCCT GATGTA
		SpCas9_OTS 1_R	CTCTTCTGCTGGAT TGGTTC
SpCas9_ OTS 2	CA- AGtGTCCgTGGTGGCCAAG G	SpCas9_OTS 2_F	TCTGTAAGCATCTC TCCCTG
		SpCas9_OTS 2_R	TTCAAATGCCTCTG ACAAAT
SpCas9_ OTS 3	CATAGgGTtCTgGGTGGCCA GGG	SpCas9_OTS 3_F	AGTACCTTGCCAGT AAGTGAC
		SpCas9_OTS 3_R	GTTCTTTTCCATAGC ACTGAC
SpCas9_ OTS 4	CATAGtcTCCTTGGTcGCCAA GG	SpCas9_OTS 4_F	CAAAGGAGTGAGAT GTGTAT
		SpCas9_OTS 4_R	GTCAAGGTGTAGAA AAGAGA

Table S5

**Targets**	**Guide_sequence**	**PCR_primer_name**	**Sequence (5'- 3')**	**Nuclease**	**PAM**
HEK293T _ADRB2	TGCCTGTATGTG CGTGCCTCGGT	HEK293T_ADRB2_ seq_F	GGAGGGTG TGTCTCAG TGTC	**Cas12i**	**TTG**
	TGCCTGTATGTG CGTGCCTCGGT	HEK293T_ADRB2_ seq_R	GCTTTTGG CTCTTCTGT GGC	**LbCas12a**	**TTT G**
				**SpCas9**	**TG**

	CCTGTATGTGCGTGCCTCGG				**G**
HEK293T_CHRM4	TCTCCCCTTCCTCCCCAGACACG	HEK293T_CHRM4_seq_F	ATTCTGCCAGAGAATGTCCCTC	**Cas12i**	**TTC**
	TCTCCCCTTCCTCCCCAGACACG	HEK293T_CHRM4_seq_R	ATTTCCACCGTCTCATAGCGA	**LbCas12a**	**TTTC**
	CTCCCCTTCCTCCCCAGACA			**SpCas9**	**CGG**
HEK293T_CXCR4	AGGGAAGCGGGATGCGCCTGAAG	HEK293T_CXCR4_seq_F	CCTGGGCCTCAGTGTCTCTA	**Cas12i**	**TTG**
	AGGGAAGCGGGATGCGCCTGAAG	HEK293T_CXCR4_seq_R	CAGGGGACCCTGCTGTTTG	**LbCas12a**	**TTTG**
	AGCGGGATGCGCCTGAAGAC			**SpCas9**	**AGG**
HEK293T_FANCF	CCCAGAAGCCAGTGGACTAGCAC	HEK293T_FANCF_seq_F	GTTTCTCAGCAGCCTGTGGGA	**Cas12i**	**TTC**
	GAAGCCAGTGGACTAGCACT	HEK293T_FANCF_seq_R	GGGTGGCGGCTAGTCACTAAAG	**SpCas9**	**TGG**
HEK293T_AR	CTCTAGGAACCCTCAGCCCCAAG	HEK293T_AR_seq_F	TCTGGACAAAATTGAGCGCC	**Cas12i**	**TTG**
	CTCTAGGAACCCTCAGCCCCAAG	HEK293T_AR_seq_R	CGTCATAGGGATAGATCGGGC	**LbCas12a**	**TTTG**
	CCCTCAGCCCCAAGAATCAG			**SpCas9**	**AGG**
HEK293T_CD2	CCTATATTTCTATGTGGTCTTGT	HEK293T_CD2_seq_F	TCCTCATCATTTTATGGGTCTACA	**Cas12i**	**TTA**
	CCTATATTTCTATGTGGTCTTGT	HEK293T_CD2_seq_R	AGAGAAAAGGAACAGAGAGGCT	**LbCas12a**	**TTTA**
	TATTTCTATGTGGTCTTGTT			**SpCas9**	**AGG**
HEK293T_HBB	GTAGCAATTTGTACTGATGGTAT	HEK293T_HBB_seq_F	TGCTTACCAAGCTGTGATTCCA	**Cas12i**	**TTA**
	GTAGCAATTTGT	HEK293T_HBB_se	GCTCCTGG	**LbCas12a**	**TTT**

	ACTGATGGTAT	q_R	GAGTAGATTGGC		**A**
	AGCAATTTGTACTGATGGTA			**SpCas9**	**TGG**
HEK293T_GRIN2B	GTGCTCAATGAAAGGAGATAAGG	HEK293T_GRIN2B_seq_F	TCTCATTCTGCAGAGCAAATACC	**Cas12i**	**TTG**
	GTGCTCAATGAAAGGAGATAAGG	HEK293T_GRIN2B_seq_R	GGAGAACAGCACTCCGCTC	**LbCas12a**	**TTTG**
	GTGCTCAATGAAAGGAGATA			**SpCas9**	**AGG**

Table S6

**Targets**	**Guide_sequence**	**PCR_primer_name**	**Sequence (5'- 3')**	**Position in the array**
ACTB_1	CGGATGTCCACGTCACACTTCAT			**1**
ACTB_2	CCTCTCAGGCATGGAGTCCTGTG			**2**
GAPDH_1	GCTACAGCAACAGGGTGGTGGAC	Array_3_GAPDH_T7E1_F	CGGGAAACTGTGGCGTGAT	**3**
		Array_3_GAPDH_T7E1_R	GGTCTACATGGCAACTGTGA	
GAPDH_2	AACAGCGACACCCACTCCTCCAC			**4**
LMNA_1	GAGCCCCCAGAACTGCAGCATCA	Array_5_LMNA_T7E1_F	TCCCTGGTCATCGAGGGGTA	**5**
		Array_5_LMNA_T7E1_R	TGGAATCACAGCAGGCCAAG	
LMNA_2	AGGGTGAACTTTGGTGGGAACCG			**6**
AR	CTCTAGGAACCCTCAGCCCCAAG	Array_7_AR_T7E1_F	CCTTTACACAGGGCTTATGG	**7**
		Array_7_AR_T7E1_R	CCAGGGGTTCTTTTCAGGAC	
ADRB2	TGCCTGTATGTGCGTGCCTCGGT	Array_8_ADRB2_T7E1_F	GGGAGGGGAGCATTATCAGT	**8**
		Array_8_ADRB2_T7E1_R	GCTAAAGCTGGAGGTGGTGT	
CCR4	GCACATCTTCTTG	Array_9_CCR4_T	CCTGCATATCCATG	**9**

	GCCTTCGGTC	7E1_F	ATGAGA	
		Array_9_CCR4_T7E1_R	CTTCCTGTGAGCACGAGTTA	
CCR10_1	GTAAAATCCAGATCCTGCACAAG			**10**
CCR10_2	TTCACTGACAAAGACAGTAAATT			**11**
CD2	CCTATATTTCTATGTGGTCTTGT	Array_12_CD2_T7E1_F	TTTCTCTCCCTGTTCCCTCT	**12**
		Array_12_CD2_T7E1_R	ATGTCCAAGTTGATGTCCTG	
CHRM4_1	TCTCCCCTTCCTCCCCAGACACG			**13**
CHRM4_2	CATGTCTCCCCCCATGAGGCAGG			**14**
CXCR4_1	GCAAGGATGGACGCGCCACAGAG			**15**
CXCR4_2	AGGGAAGCGGGATGCGCCTGAAG			**16**
HBB_1	AAGTCCAACTCCTAAGCCAGTGC			**17**
HBB_2	GTAGCAATTTGTACTGATGGTAT			**18**
IL1RN_1	GGTAAGCTCCTTCCACTCTCATT			**19**
IL1RN_2	CAGGAGGGTGACTCAGGCTAGCA			**20**
DNMT1_1	GGGTGTTCTGTCACAGAAGACAA	Array_21_DNMT1_T7E1_F	ACTGACTGACGCTGATCGCAC	**21**
		Array_21_DNMT1_T7E1_R	CGGGTCAGAAGTGAGGGTGCT	
DNMT1_2	TTCTGCAGGAAGCGGTCTAGCAA			**22**
DNMT1_3	CTGATGGTCCATGTCTGTTACTC			**23**
EMX1_1	CCATAGAGTCCTTGGTGGCCAAG	Array_24_EMX1_T7E1_F	CTCTGAGAACTGAAACGACATC	**24**
		Array_24_EMX1_T7E1_R	GGGTAGAAATGGAGAGGGTC	
EMX1_2	TGGTTGCCCACCCTAGTCATTGG			**25**
FANCF_1	CCCAGAAGCCAGTGGACTAGCAC			**26**

FANCF_2	GGTTCTCTCTATA GCCATTGAAG			**27**
FANCF_3	GGCGGGGTCCA GTTCCGGGATTA	Array_28_FANCF _T7E1_F	TGCCCAGAGTCAA GGAACAC	**28**
		Array_28_FANCF _T7E1_R	CCAGGCTCTCTTG GAGTGTC	
GRIN2B	GTGCTCAATGAA AGGAGATAAGG			**29**
VEGFA	CTAGGAATATTGA AGGGGGCAGG	Array_30_VEGFA _T7E1_F	GTGAGCCTGGAGA AGTAGCC	**30**
		Array_30_VEGFA _T7E1_R	GTGATTTGGGGAA GTAGAGCA	
